# Use of Optical Genome Mapping to Detect Structural Variants in Neuroblastoma

**DOI:** 10.3390/cancers15215233

**Published:** 2023-10-31

**Authors:** Ruby G. Barford, Emily Whittle, Laura Weir, Fang Chyi Fong, Angharad Goodman, Hannah E. Hartley, Lisa M. Allinson, Deborah A. Tweddle

**Affiliations:** 1 Wolfson Childhood Cancer Research Centre, Translational & Clinical Research Institute, Newcastle University Centre for Cancer, Newcastle University, Newcastle upon Tyne NE1 7RU, UK; ruby.barford@newcastle.ac.uk (R.G.B.); f.c.fong2@newcastle.ac.uk (F.C.F.); hannah.hartley@newcastle.ac.uk (H.E.H.); lisa.allinson@newcastle.ac.uk (L.M.A.); 2 Newcastle Genetics Laboratory, Newcastle upon Tyne Hospitals NHS Trust, Newcastle upon Tyne NE1 3BZ, UK; emily.whittle2@nhs.net (E.W.); angharad.goodman1@nhs.net (A.G.); 3 Great North Children’s Hospital, Newcastle upon Tyne NE1 4LP, UK

**Keywords:** optical genome mapping, neuroblastoma, MYCN, chromothripsis, chromoplexy

## Abstract

**Simple Summary:**

Copy number abnormalities (CNAs) are frequent in neuroblastoma and used to determine treatments. Little is known about the role of structural variants (SVs) in disease progression. Optical genome mapping (OGM) uses label patterns to scan the genome which can be used to identify SVs in cancer. Currently, the detection of SVs still relies on standard cytogenetic techniques. We investigated the utility of OGM to detect SVs in neuroblastoma cell lines and tumours and compared it with standard cytogenetic techniques. OGM confirmed known CNAs and identified novel SVs of potential clinical significance.

**Abstract:**

Background: Neuroblastoma is the most common extracranial solid tumour in children, accounting for 15% of paediatric cancer deaths. Multiple genetic abnormalities have been identified as prognostically significant in neuroblastoma patients. Optical genome mapping (OGM) is a novel cytogenetic technique used to detect structural variants, which has not previously been tested in neuroblastoma. We used OGM to identify copy number and structural variants (SVs) in neuroblastoma which may have been missed by standard cytogenetic techniques. Methods: Five neuroblastoma cell lines (SH-SY5Y, NBLW, GI-ME-N, NB1691 and SK-N-BE2(C)) and two neuroblastoma tumours were analysed using OGM with the Bionano Saphyr^®^ instrument. The results were analysed using Bionano Access software and compared to previous genetic analyses including G-band karyotyping, FISH (fluorescent in situ hybridisation), single-nucleotide polymorphism (SNP) array and RNA fusion panels for cell lines, and SNP arrays and whole genome sequencing (WGS) for tumours. Results: OGM detected copy number abnormalities found using previous methods and provided estimates for absolute copy numbers of amplified genes. OGM identified novel SVs, including fusion genes in two cell lines of potential clinical significance. Conclusions: OGM can reliably detect clinically significant structural and copy number variations in a single test. OGM may prove to be more time- and cost-effective than current standard cytogenetic techniques for neuroblastoma.

## 1. Introduction

Neuroblastoma (NB) is the most frequent extracranial solid tumour in children under 15 years of age, with a median age of diagnosis of 18 months [1,2]. NB accounts for 8–10% of all paediatric tumours [3] but around 15% of all paediatric cancer deaths [1]. NB originates from the sympathoadrenal lineage of undifferentiated neural crest cells. Tumours can develop anywhere along the sympathetic nervous system; the most common primary sites are the adrenal medulla and paraspinal ganglia [4], with over 50% of primary tumours arising in the adrenal medulla [2]. NB is grouped into three risk groups, low, intermediate and high, on the basis of clinical (age and tumour spread) and biological features (including the presence of *MYCN* oncogene amplification) with treatment varying accordingly [5]. The International Neuroblastoma Staging System (INSS) is a previously used post-surgical staging system. Patients are staged from 1 to 4, with stage 1 referring to a completely resected, localised tumour and stage 4 referring to a tumour with distant metastatic spread [6]. 

There are many recurrent genetic aberrations in NB, arguably the most important being amplification of the *MYCN* oncogene, which has been reported in around 25% of NB and classifies tumours as high-risk [2,7,8]. *MYCN* is an oncogene encoding the MYCN protein which regulates the transcription of genes involved in tumorigenesis. Other common single-gene abnormalities include aberrations involving anaplastic lymphoma kinase (ALK), a receptor tyrosine kinase. *ALK* point mutations resulting in constitutive activation are found in 8–10% of NB [9,10] and gene amplification in a further 2–3% (almost exclusively with *MYCN* co-amplification) [11], leading to increased ALK activity. In relapsed NB, there is an increased frequency of mutations in genes in the RAS-MAPK pathway, including *ALK*, suggesting important roles in relapse [12]. The transformation of progenitor cells into NB has been found to be accelerated by the coexistence of *ALK* mutations and *MYCN* amplification [1].

Copy number abnormalities (CNA) are common in NB. There are seven typical segmental chromosomal alterations (SCAs) in NB, as identified by the SIOPEN group (International Society of Paediatric Oncology European Neuroblastoma Group). These are copy number gains at 1q, 2p and 17q, and copy number losses at 1p, 3p, 4p and 11q [13]. These SCAs are frequently seen in patients with a poor outcome and are associated with a higher risk of relapse [13]. In contrast, whole chromosomal aneuploidies (WCAs) without the presence of SCAs are associated with a good prognosis in NB [14].

The use of genome sequencing has given rise to the recent identification of genetic aberrations such as massive chromosomal rearrangements that occur during a single catastrophic event in the cell cycle [15]. These complex rearrangements include chromothripsis and chromoplexy. Chromothripsis is the localised shredding of chromosomes, where during a single catastrophic event, multiple double stranded breaks occur and DNA fragments are reassembled through non-homologous end joining (NHEJ) in a random order and orientation, causing tens to hundreds of intrachromosomal genomic rearrangements and forming complex derivative chromosomes [8,15,16]. Chromothripsis is seen in at least 2–3% of all cancer types [16] and was found in 18% of NB in one study, all being at INSS stages 3 and 4 [8]. In NB, chromothripsis is frequently associated with the amplification of *MYCN* or *CDK4* and the loss of the heterozygosity (LOH) of chromosome 1p. Chromothripsis has distinct characteristics, such as numerous clustered chromosomal breakpoints, it has no LOH in the affected chromosomes [15], and it is linked with oncogene amplification [17]. NB with chromothripsis has been found to have a poor prognosis [8]. Chromoplexy involves the presence of balanced translocations across up to eight chromosomes, with deletions frequently occurring at the translocation breakpoints [15,18]. Like chromothripsis, the random breakages and fusions are mediated by NHEJ [19]. Whilst chromothripsis occurs in a single catastrophic event, it is thought that chromoplexy can occur multiple times during cancer evolution [19]. These major genomic aberrations influence cancer development through the disruption of tumour suppressor genes or amplification of oncogenes [20,21].

Telomeres are repetitive regions of tandem repeat overhang, 3′-TTAGGG-5′, that form a T-loop at the end of chromosomes [22], which protects chromosomes and limits the replicative life span of somatic cells. Cellular proliferation leads to shortening of telomere lengths, and once past a threshold, cells are incapable of dividing and become senescent [22,23]. Cancer cells can survive this cellular crisis using telomere maintenance mechanisms (TMMs), which involve extending the telomere length, leading to uncontrolled proliferation. Telomere lengths are maintained using two distinct mechanisms. The first and most common is the telomerase-dependent pathway which involves the activation of the telomerase complex through increased expression of the *TERT* gene. This can happen by amplification of *TERT* or *MYCN* (a known transcriptional activator of *TERT*), structural variants (SVs) affecting *TERT*, mutations in the promoter of *TERT* or *TERT* epigenetic changes [10,24]. The telomerase complex extends the lengths of telomeres, which stops cellular senescence, maintaining tumour propagation and unlimited cellular proliferation. The second mechanism for telomere length maintenance is telomerase-independent and known as alternative lengthening of telomeres (ALT), where DNA is replicated through homologous repair using telomere DNA as a template [24,25]. The alpha thalassemia/mental retardation syndrome X-linked (*ATRX*) gene and its partner, death associated protein 6 (*DAXX*) gene, encode proteins which incorporate the histone variant H3.3 into telomeric pericentric chromatin [26]. ATRX is known to act as an ALT suppressor, and mutations in *ATRX* have been shown to be associated with maintained telomere lengths in NB [26]. One study showed that most high-risk NB tumours have *TERT* rearrangements, *MYCN* amplification or *ATRX* mutations, all of which extend telomere length [10], suggesting an important role for TMM in clinical prognosis. 

Chromosomal aberrations such as SVs are major drivers in tumorigenesis. SV detection is still dependent on standard cytogenetic techniques, but each has its limitations. For example, karyotyping has a very low resolution of around 5–10 Mb [27], and FISH has a resolution of between 100 kb and 1 Mb [28]. Optical genome mapping (OGM) images long, linear DNA molecules (>250 kb) labelled at specific base pair sequence motifs, allowing for a direct visualisation of the genome [27,29]. This has the potential to be a cost- and time-effective alternative cytogenetic technique which shortens turnaround time and reveals more clinically relevant SVs at a higher resolution than traditional cytogenetic techniques. OGM detects SVs across the genome at allele frequencies as low as 1% with sensitivities as high as 99% [30]. This enables the viewing of genomic aberrations at a 10,000× greater sensitivity than with karyotyping [29]. OGM detects SVs in an unbiased manner at higher sensitivities than other sequence-based technologies [31]. As over half of the human genome is repetitive [32], it is largely inaccessible by short-read sequencing techniques. Due to the workflow of OGM using around 500,000 fluorescent labels, changes in patterning or spacing of labels is detected automatically, elucidating the complex rearrangements found in cancer. 

OGM has been used to evaluate chromosomal aberrations in a wide range of cancers, from myeloid and lymphoid haematological malignancies to solid malignancies such as triple negative breast tumours and acral melanomas. It has recently been used to detect a novel fusion in an adult AML patient [33], and in metastatic lung squamous cell carcinomas, OGM was found to identify large SVs not detected by whole genome sequencing (WGS), which may be important in disease progression [34]. In this study we used OGM to detect SVs in five neuroblastoma cell lines and two tumour samples. We compared SV detection using OGM to standard cytogenetic methods for the cell lines, as well as to pre-existing WGS data for the tumours. Here, we show that OGM can confirm known CNAs and detect novel SVs of potential clinical significance.

## 2. Methods

### 2.1. Cell Lines and Tumour Samples

#### 2.1.1. Cell Lines

Five human NB cell lines were selected with a variety of known genetic abnormalities including *MYCN* (and other oncogene amplifications) together with other TMMs such as *TERT* rearrangement (Table 1).

#### 2.1.2. Tumour Samples

NB tumour samples which had undergone SNP array and WGS were obtained from the VIVO biobank following approval by the Children’s Cancer & Leukaemia Group (CCLG) biological studies steering group (CCLG 2016-02 and CCLG 2019-01) (Table 2). 

### 2.2. Cell Culture

Cell lines were cultured in an RPMI-1640 culture medium (Sigma-Aldrich, St. Louis, MO, USA) with 10% foetal bovine serum (FBS) (Thermo Fisher Scientific Inc., Waltham, MA, USA) at 37 °C in 5% CO_2_. Cells were tested and found to be free from Mycoplasma. Cells were passaged when 70–80% confluent by washing with phosphate-buffered saline (PBS) and adding 1× trypsin-EDTA (Sigma-Aldrich, St. Louis, MO, USA) in PBS. RPMI with 10% FBS was added to neutralise the trypsin-EDTA, and the cell solution was centrifuged for 5 min at 1200 rpm. The supernatant was removed leaving cells to be harvested or resuspended in the media for continued culture. When harvesting, a 70–80% confluent T75 flask of cells was frozen at −80 °C before DNA and RNA extraction.

### 2.3. RNA Fusion Panel

RNA fusion panel analysis was carried out on all five NB cell lines. Total RNA extraction from cell pellets was carried out using the AllPrep DNA/RNA Mini Kits (QIAGEN, Germantown, MD, USA), according to the manufacturer’s instructions [44]. Extracted RNA was initially quantified using the Agilent Bioanalyzer RNA Nano 6000 kit (Agilent Technologies Inc., Santa Clara, CA, USA), providing a DV200 score that indicates total RNA quality. A threshold DV200 score > 30% was applied. The samples were scanned using the NextSeq 550 System (Illumina Inc., San Diego, CA, USA). The Illumina TruSight RNA Fusion Panel (Illumina Inc., San Diego, CA, USA) was used to detect comprehensive gene fusions in NB cell lines. There are 507 identifiable genes in the fusion panel (Supplementary Table S1), and any fusion involving these genes will be detected, regardless of the partner gene. Fusion panel analysis was carried out according to the Illumina standard operating procedure [45].

### 2.4. Whole Genome Sequencing (WGS)

WGS was carried out on tumour samples as part of the NHS Genomic Medicine Service. DNA extraction from the tumours was carried out by the Newcastle NHS Genetics Laboratory and sent to the centralised NHS Genomics Medicine Sequencing Centre at the Wellcome Genome Campus, Hinxton, Cambridge. Tumour sequence data were put through Genomics England Cancer Pipelines v2.15 and v2.27. The sequence was aligned to the GRCh38 reference using DRAGEN aligner (v3.2.22) and included alternate haplotypes (ALT contigs), which were stored as CRAM files. Quality metrics were calculated using SAMtools. Cross-patient contamination was kept at ≤1% in tumour samples using the ConPair algorithm. Copy number and SVs were reported using Canvas (v1.39), Manta (v1.5) and PINDEL (v0.2.5b9), with copy-neutral SVs such as inversions and translocations called exclusively by Manta using default settings. Manta-called SVs were excluded when the depth in the germline sample near one or both breakpoints was 3× higher than the chromosomal median, or when the somatic quality score was <30. Only variants > 1 kb were examined Artefacts were filtered out after inspection by the Integrated Genome Viewer. Only Manta calls in protein-coding regions, as well as immunoglobulin and T-cell receptor loci, were reported.

### 2.5. Single-Nucleotide Polymorphism Array

SNP arrays were performed on all cell lines and tumour samples using Infinium CytoSNP-850Kv1.1 and CytoSNP-850Kv1.2 BeadChip arrays according to the manufacturer’s instructions (Illumina Inc., San Diego, CA, USA). The BeadChips were scanned using the NextSeq 550 System (Illumina Inc., San Diego, CA, USA), and configured data in IDAT files were subsequently inputted into Genome Studio 2.0 software (Illumina Inc., San Diego, CA, USA). The output was viewed using Nexus Copy Number v10.0 software (Bionano Genomics Inc., San Diego, CA, USA). Only copy number variants (CNVs) over 3 Mb were called unless focal regions with isolated high copy number gains were found. CNV coordinates were recorded at a resolution of 0.01 Mb. Cut-offs to call CNVs were scored using the criteria set by Depuydt et al. [46]. Illumina cut offs (log2 ratio): Chromosomal gain ≥ +0.15, chromosomal loss ≤ −0.25, gene amplification > +0.7, homozygous loss < −2. Chromothripsis was defined as >10 oscillating copy numbers on a chromosome. 

### 2.6. TERT Fluorescent In Situ Hybridisation 

A quantity of 4 µL of *TERT* BreakApart probe (Cytocell, Cambridge, UK) was diluted 1:1 with hybridisation buffer (Thermo Fisher Scientific Inc., Waltham, MA, USA) and added to a glass slide. An 8 mm cover slip was added, sealed by Fixogum (Marabu, Tamm, Germany) and hybridised at 72 °C for 5 min. Slides were washed and stained using Vectashield Antifade Mounting Medium with the fluorescent stain DAPI (Vector Laboratories Inc., Newark, CA, USA). A 24 mm × 60 mm cover slip (DURAN Group GmbH, Mainz, Germany) was applied to the slide, which was kept in the dark until scored. 

On each slide, 100 cells were scored with a Leica DM5500B microscope (Leica Microsystems GmbH, Wetzlar, Germany). The cut-off was previously determined using three control samples of healthy bone marrow. Slides were subsequently scored by two other scientists. 

### 2.7. Optical Genome Mapping

All OGM procedures were performed following the manufacturer’s guidelines (Bionano Genomics Inc., San Diego, CA, USA).

#### 2.7.1. Isolation of UHMW DNA

Ultra-high molecular weight (UHMW) genomic DNA (gDNA) was extracted from samples using the Bionano SP Blood and Cell DNA Isolation Kit according to the protocol (Bionano Prep SP Frozen Cell Pellet DNA Isolation Protocol v2 (30398, Rev B) for cell lines and Bionano Prep SP Tissue and Tumour DNA Isolation Protocol (30339, Rev A) for tumour samples). For cell lines, DNA was isolated from cell pellets containing 1.5 million cells. For tumour samples, the tumour tissue was cut into 10 mg slices. The tissue was homogenised using a TissueRuptor (QIAGEN, Germantown, MD, USA) and washed with 6 mL ice-cold Homogenization Buffer (Bionano Genomics Inc., San Diego, CA, USA). Cells were lysed and digested using proteinase K, RNase K and Lysis and Binding Buffer. Then, 100 mM phenylmethylsulfonyl fluoride (PMSF) was added to inactivate lysis and prevent DNA molecules from being too short. gDNA was precipitated with isopropanol and bound to a Nanobind disk (Pacific Biosciences of California Inc., Menlo Park, CA, USA) for washing. The samples were left overnight at room temperature to homogenise and then quantified on the Qubit Fluorometer using the Qubit dsDNA Broad Range Assay Kit (Thermo Fisher Scientific, Waltham, MA, USA). DNA concentrations of 50–120 ng/μL were selected to ensure adequate size and homogeneity.

#### 2.7.2. Labelling of UHMW gDNA 

gDNA was labelled using the Direct Label and Stain (DLS) technique following the Bionano Prep Direct Label and Stain (DLS) Protocol (30206, Rev G). Briefly, Direct Labelling Enzyme (DLE-1) was added to 750 ng of DNA from each sample to label the DNA at specific 6 bp-sequence motifs, CTTAAG, which occur 15 times per 100 kbp [30], with DL-green fluorophores. Proteinase K was added for digestion. DL-green clean-up was performed using a two-step DLS membrane and adsorption microplate to eliminate systematic DNA molecule breaks and prevent DNA repair. After washing out the green fluorophore excess, the DNA was left overnight to ensure counterstaining of its backbone. DNA quantification was performed on the Qubit Fluorometer using the Qubit dsDNA High-Sensitivity Assay Kit (Thermo Fisher Scientific, Waltham, MA, USA) to ensure that the concentration fell between 4 and 12 ng/μL.

#### 2.7.3. Data Collection

Labelled gDNA samples of the correct concentration were loaded onto the Bionano Saphyr chip and imaged with the Saphyr instrument. Gene coverage was targeted at 1500×. The pipelines were run using DLE-1 labelled human genome as a reference (hg19_DLE1_0kb_0labels.cmap).

#### 2.7.4. OGM Data Analysis

OGM data analysis was performed using the de novo pipeline for cell lines and the rare variant pipeline for tumour samples, both included in the Bionano Solve software (v3.7_20221013_25). Data were directly visualised in Bionano Access software (v1.7.2) and used the human genome GRCh37/Hg19 as a reference. All filters were set according to the recommendations from Bionano (Supplementary Table S2). The self-molecule count was set to 5. Results were analysed through two pipelines: CNV pipeline and SV pipeline. The CNV pipeline allows for the detection of large, unbalanced aberrations, and the SV pipeline compares the labelling patterns between the sample genome map and the reference genome map. SV and CNV were identified through differences in alignments between the reference genome and sample genome map. The following confidence scores were applied as recommended by the manufacturer: insertions/deletions ≥ 0, inversion ≥ 0.7, duplication ≥ −1, translocation/intrachromosomal fusion ≥ 0.05, copy number ≥ 0.99 and aneuploidy changes ≥ 0.95. The ‘hg19 Known Canonical Mapped’ feature file was added on Bionano Access software (v1.7.2).

## 3. Results

### 3.1. DNA Molecule Quality Report 

The molecule quality report (MQR) provided by Bionano Access^®^ software with each analysis shows a summary of the DNA molecule quality based on the molecule-to-reference alignment results per sample (Table 3). The data were classified as satisfactory or unsatisfactory based on recommendations from Bionano. Only samples meeting at least one of these criteria were included in the final analyses.

### 3.2. Comparison of OGM of NB Cell Lines with Other Cytogenetic Analyses

#### 3.2.1. NB1691 Cell Line

**Figure 1:**
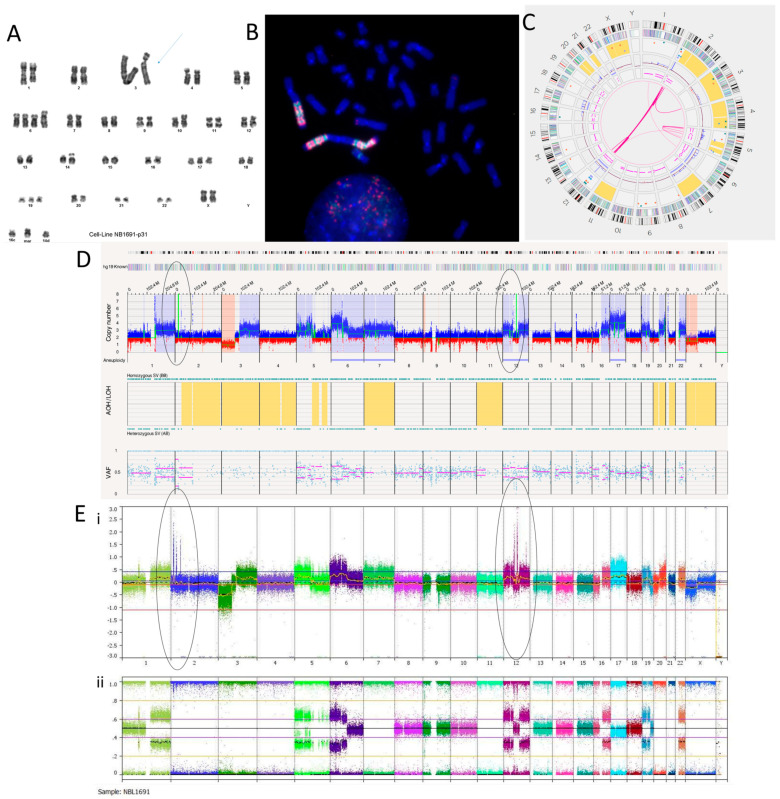
NB1691 neuroblastoma cell line. (**A**) Karyotype showing homogenously stained regions (HSRs) on chromosome 3 (arrow). (**B**) Metaphase FISH analysis. *MYCN* probe fluorescently labelled red. *MDM2* probe fluorescently labelled green. Three HSRs are on chromosome 3, concordant with the karyotype. (**C**) OGM Circos plot view showing chromoplexy on chromosomes 2 and 12 and hyper-rearrangement on chromosome 5. (**D**) OGM whole genome view. OGM SV/SNV Key: Green, insertion; Orange, deletion; Light blue, inversion; Purple, duplication; Pink, Intra-chromosomal-fusion or Inter-chromosomal-Translocation; Yellow, LOH region; Dark blue, CNV Gain; Red, CNV Loss. (**E**) SNP array analysed using Nexus software. (**i**) log2 ratio (**ii**) B allele frequency. Amplicons on chromosomes 2 and 12 highlighted in (**D**,**E**) by ellipses.

We have recently reported the NB1691 cell line to be *MYCN*, *ALK* and *MDM2* amplified [36]. The karyotyping of the NB1691 neuroblastoma cell line showed a complex tumour clone with a chromosome count of 51–54 chromosomes (Figure 1A). The presence of homogenously staining regions (HSRs) on chromosome 3 shown on the karyotype suggests that these regions were amplified. This was confirmed by the fluorescence in situ hybridisation (FISH) of metaphase spreads showing HSRs with a clear hybridisation of both the *MDM2* and the *MYCN* probes (Figure 1B). The SNP array analysis reported amplification at 2p24.3 and 12q15 coinciding with the *MYCN* region and *MDM2* genes, respectively (Figure 1E).

The Circos plot from OGM suggests that chromoplexy occurs between chromosomes 2 and 12 due to the presence of many translocations between the same breakpoints (Figure 1C). Other SVs, such as deletions, duplications and inversions, can be seen at these breakpoints (Supplementary Table S3). Large amplicons also occur here, which is consistent with the SNP array and FISH findings.

The amplicon located on chromosome 2p24.3 locus has a reported copy number between 60 and 100 (Figure 1D). This amplicon overlaps with genes *BC035112*, *FAM84A*, *AX747684*, *NBAS*, *DDX1*, *AK093525*, *MYCN*, *MYCNOS*, *SNORA40* and *FAM49A*. *MYCN* has a copy number estimated at 87.33. The amplicon located on the chromosome 12q15 locus has a reported copy number between 30 and 120. It overlaps with *MDM2* and other genes. *MDM2* has a copy number called between 70 and 100 and overlaps with two inverted duplications. They are both 160,259 bp long and overlap with the genes *SLC35E3*, *LOC100130075*, *MDM2* and *CPM*. A putative fusion gene, *SLC35E3-CPM*, was found as a result of these inverted duplications, but it did not produce a fusion transcript with the RNA fusion panel. Another amplicon located on chromosome 12 over the loci q13.3 to q14.1 includes *CDK4* with a copy number estimated at 64.29 (Figure 1D,E). Within this amplicon are many translocations and intra-chromosomal fusions, along with other SVs such as inversions, duplications, deletions and insertions. 

Out of the 47 translocations occurring in the NB1691 cell line (Supplementary Table S3), 41 are between chromosomes 2 and 12, suggesting that chromoplexy is occurring between these chromosomes. Furthermore, 22 intra-chromosomal fusions are present on chromosome 12. This suggests chromosome 12 chromothripsis. Alternations in copy number states can be seen on the SNP array (Figure 1E), which meets the cut-off confirming chromothripsis. There are six intra-chromosomal fusions occurring on chromosome 5, which is not enough to confirm chromothripsis but is instead classed as hyper-rearrangement. This is confirmed on the SNP array, where <10 oscillations in copy number states can be seen (Figure 1E). The overall copy number profiles obtained by OGM and the SNP array were highly concordant (Figure 1D,E, Table 4).

Twelve translocations and two intra-chromosomal fusions overlap with the *ALK* gene. *ALK* is located at the chromosome 2p23.2 locus and has a copy number called at ~20. We previously reported *ALK* as amplified in the NB1691 cell line by SNP array [36]. Translocations and inversions are likely to cause constitutive activation of ALK.

OGM found a putative gene fusion between *PTPRR* and *BEST3* around 1,091,696 bp long, caused by multiple deletions mapping to the chromosome 12q15 locus. This fusion is not in-frame but includes *PTPRR* exons 1–5 and *BEST3* exon 10. The essential catalytic site for PTPRR is a protein tyrosine phosphatase catalytic domain (PTP) coded in exon 13, which would not be translated, resulting in a truncated *PTPRR* gene and loss of function of the protein. An RNA fusion panel confirmed a putative gene fusion between *PTPRR* and *BEST3* (Supplementary Table S4). There were no other fusions detected by the RNA fusion panel in this cell line.

#### 3.2.2. SH-SY5Y Cell Line

**Figure 2:**
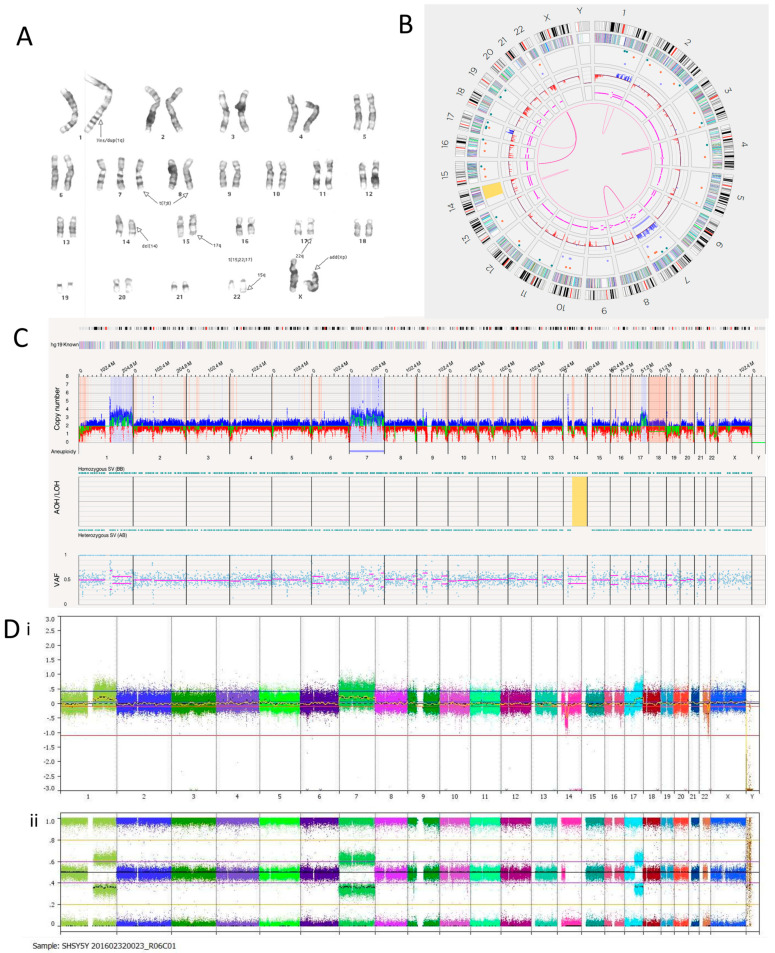
SH-SY5Y neuroblastoma cell line. (**A**) Karyotype. The representative composite karyotype was determined to be: 47, X, add(X)(p22), ins(1)(q1) or dup(1)(q?25;q?32),+7, t(7;8)(q3?2;q24), del(14)(q13q22), t(15;22;17)(q22-24;q13;q12). (**B**) OGM Circos plot view. (**C**) OGM whole genome view. OGM SV/CNV Key: Green, insertion; Orange, deletion; Light blue, inversion; Purple, duplication; Pink, Intra-chromosomal fusion or Inter-chromosomal Translocation; Yellow, LOH region; Dark blue, CNV Gain; Red, CNV Loss. (**D**) SNP array analysed using Nexus software. (**i**) log2 ratio (**ii**) B allele frequency.

A karyotype previously carried out on the SH-SY5Y cell line in our laboratory suggested additional chromosomal material on the p-arm of chromosome X, insertion or duplication on chromosome 1, trisomy of chromosome 7, and a deletion on chromosome 14. It also showed a translocation between chromosomes 7 and 8 and a three-way translocation between chromosomes 15, 22 and 17 but did not identify the breakpoints (Figure 2A). OGM confirmed the trisomy of chromosome 7, with an estimated copy number of 3.054 (Figure 2B). OGM did not detect structural variants or copy number gain involving the p-arm of chromosome X. No deletion was detected on chromosome 14, although copy number loss and loss of heterozygosity were observed on 14q (Figure 2B,C). The karyotype was carried out 15 years ago; therefore, these discrepancies could be a result of multiple passages of the cell line since then. For all cell lines, OGM was carried out within six passages of receipt and within two passages of the SNP array (Figure 2D). Despite this, SH-SY5Y showed clear evidence of genetic drift between the OGM and the SNP array results (Table 4)**.** OGM identified an inverted duplication on chromosome 1 at the q25.2 locus of around 266,603 bp, which overlapped with genes *ASTN1* and *FAM5B*. Three insertions were present on chromosome 1. Two were located at the q22 locus and were 527 bp long. The other was located at the q24.2 locus and was 3194 bp long. These overlapped with the *ARHGEF2* and *ADCY10* genes, respectively.

OGM identified three translocations between chromosomes 7 and 8. They mapped to t(7;8)(q33;q24.21) and overlapped with *EXOC4* and *PVT1*. An RNA fusion panel (Supplementary Table S4) confirmed a putative fusion gene between *EXOC4* exons 1–7 and *PVT1* exons 7–9, which is consistent with the OGM findings. The fusion transcript was discovered to have an ATG codon at the start of exon 8 in *EXOC4*, which could act as an alternative start site, thus producing a truncated protein if there was an alternative transcript. *PVT1* is a long non-coding RNA (lncRNA) that produces an oncogenic RNA transcript. Deregulation of this transcript has been implicated in several cancer types [47]. Focal amplification of *EXOC4* has also been previously reported in the SH-SY5Y cell line [48]. OGM confirmed this as the copy number was estimated to fall between 7 and 8. Fusion transcripts involving *PVT1* are usually characterised by an amplicon at the 8q24.21 locus [49], but this was not found in the SH-SY5Y cell line.

There were eight translocations and four intra-chromosomal fusions in the SH-SY5Y cell line (Table 5). Translocations between chromosomes 15 and 22, as well as 17 and 22, were detected using OGM in line with the three-way translocation found through karyotyping. 

The SH-SY5Y cell line is a thrice-cloned subline of the SK-N-SH cell line. The SK-N-SH cell line has been reported to have a *TP73* deletion [38]. *TP73* is a tumour suppressor gene with similar functional homologies to *TP53* located at the chromosome 1p36 locus [50]. OGM of SH-SY5Y cells reported a copy number of around 0.3, confirming the deletion and presumed loss of function. 

#### 3.2.3. GI-ME-N Cell Line

**Figure 3:**
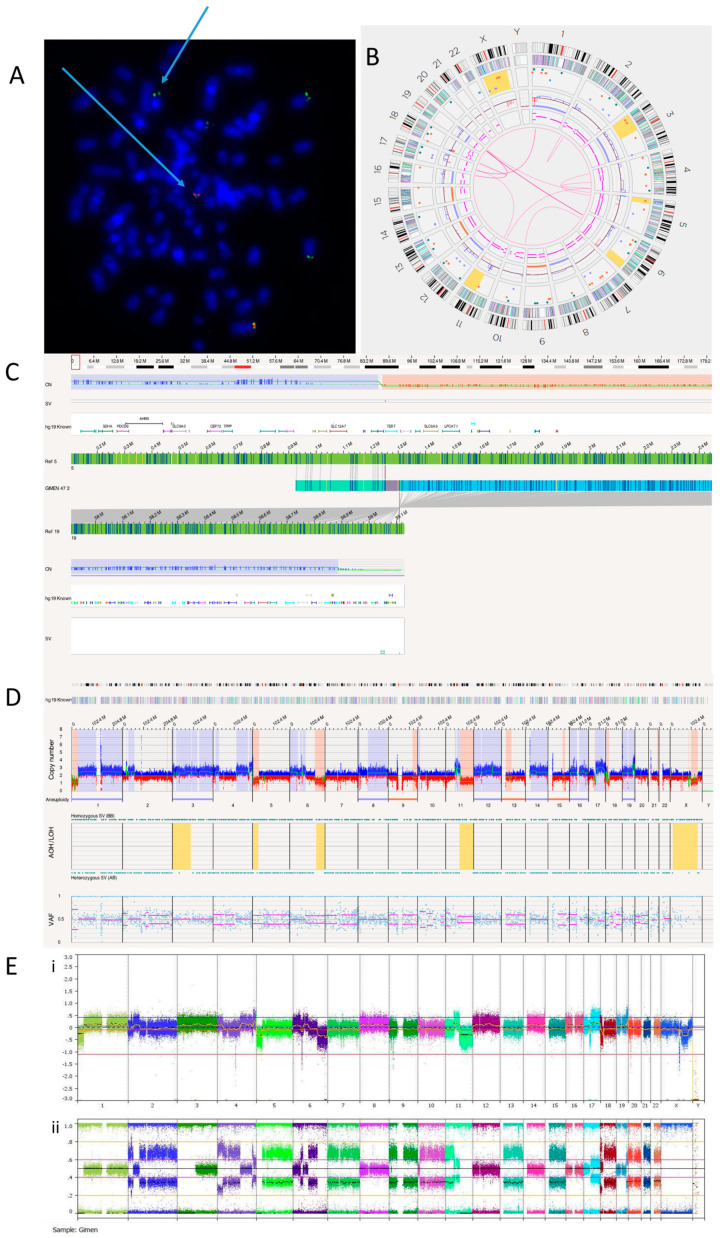
GI-M-EN neuroblastoma cell line. (**A**) *TERT* break-apart FISH showing a *TERT* rearrangement on one chromosome (arrowed). The red probe binds the centromere and the green probe the telomere. Image shows separated signals on one chromosome confirming a *TERT* rearrangement. (**B**) OGM Circos plot view. (**C**) OGM view of translocation mapped to t(5;19)(p15.33;q13.43) overlapping the *TERT* gene. Red box represents loci where SV is present. (**D**) OGM whole genome view. OGM SV/SNV Key: Green, insertion; Orange, deletion; Light blue, inversion; Purple, duplication; Pink, Intra-chromosomal fusion or Inter-chromosomal Translocation; Yellow, LOH region; Dark blue, CNV Gain; Red, CNV Loss. (**E**) SNP array analysed using Nexus software. (**i**) log2 ratio (**ii**) B allele frequency.

FISH analysis confirmed a *TERT* rearrangement at 5p15.33 (Figure 3A). OGM detected a translocation between chromosomes 5 and 19 (Figure 3B, Table 6). We identified this translocation as t(5;19)(p15.33;q13.43), with *TERT* being the nearest overlapping gene (Figure 3C)*. TERT* aberrations are almost always mutually exclusive with *ATRX* and *MYCN* defects [51]. OGM reported that *ATRX* has a copy number of 1.83 and *MYCN* a copy number of 2.6, suggesting no deletion or amplification of either gene. The *TERT* rearrangement explains the TMM positive phenotype and high *TERT* expression previously reported in GI-ME-N cells [10]. An RNA fusion panel was carried out on the GI-ME-N cell line, and no fusion was detected as *TERT* is not included in this panel (Supplementary Table S1). The whole genome views of OGM (Figure 3D) and SNP array (Figure 3E) are comparable, with both showing 5p loss, suggesting a possible *TERT* abnormality.

Table 6 shows the 13 translocations and 3 intra-chromosomal fusions present in the GI-ME-N cell line.

A previously published karyotype of the GI-ME-N cell line reported a complex tumour clone consisting of 92 chromosomes with duplications at 2p and 4q, deletions at 6p and 6q, addition at 10p and translocations between chromosomes 1 and 17, and 17 and 20 [39]. OGM was able to confirm almost all these previous analyses but was unable to detect tetraploidy (Figure 3D).

Karyotyping previously identified translocations between chromosomes 1 and 17 and chromosomes 17 and 20. OGM did not detect the translocation between chromosomes 17 and 20 but detected two translocations involving chromosome 17. These were identified as novel translocations: t(11;17)(q13.4;q12), with overlap genes *SHANK2* and *AP2B1,* and t(1;17)(p35.2;q11.2), with the overlap gene *NF1*, an important tumour suppressor previously reported to be deleted in GI-M-EN cells [52]. Consistent with this previous report, the copy number of *NF1* was found to be between 0.13 and 1.23. 

#### 3.2.4. SK-N-BE(2)C Cell Line

**Figure 4:**
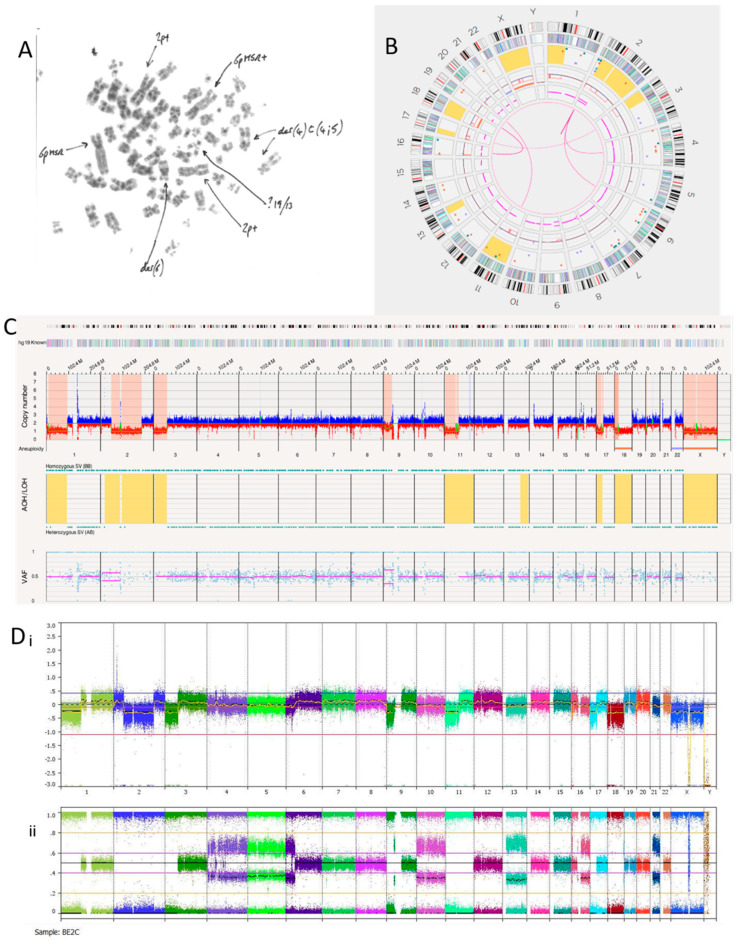
SK-N-BE(2)C neuroblastoma cell line. (**A**) Karyotype view. Major SVs are labelled with arrows. (**B**) OGM Circos plot view. (**C**) OGM whole genome view. OGM SV/SNV Key: Green, insertion; Orange, deletion; Light blue, inversion; Purple, duplication; Pink, Intra-chromosomal-fusion or Inter-chromosomal Translocation; Yellow, LOH region; Dark blue, CNV Gain; Red, CNV Loss. (**D**) SNP array analysed using Nexus software. (**i**) log2 ratio (**ii**) B allele frequency.

The SK-N-BE(2)C cell line is known to be *MYCN* amplified with a *TP53* mutation [40]. We hypothesised this cell line may, therefore, show a replicative stress signature, which OGM may detect if present. Previous karyotypes reported monosomy of chromosomes 17 and 18. A karyotype of SK-N-BE(2)C cells carried out in Newcastle Genetics Laboratory (Figure 4A) showed HSRs on chromosome 6p (indicated by the arrows in the figure). OGM detected aneuploidy loss/monosomy for chromosome 18 but not chromosome 17, possibly because of a balanced 17q gain (Figure 4B). There was a loss of heterozygosity of 17p, including the *TP53* locus, with a copy number of 1.47. Together with a *TP53* mutation, this indicates biallelic inactivation of *TP53* as we previously reported [40].

SK-N-BE(2)C cells had 11 inter-chromosomal translocations and 12 intra-chromosomal-fusions (Table 7). One translocation and all inversions occurring at the chromosome 2p24.3 locus overlapped with *MYCN*. At the chromosome 2p24.3 locus was an amplicon with a copy number between 180 and 460 (Figure 4C,D). Copy numbers of *MYCN* were reported by OGM to be between 179.04 and 186.84. Included in this amplicon are the *MYCNOS* and *SNORA40* genes. Further disruptions at this amplicon include deletions, inversions, insertions and duplications (both split and inverted), many of which overlap with *MYCN*. 

Although the SK-N-BE(2)C cell line is *MYCN* amplified with biallelic inactivation of *TP53,* the Circos plot (Figure 4B) showed a limited number of translocations and inversions, suggesting relatively low levels of replicative stress.

Copy number alteration calls for SK-N-BE(2)C cell lines were similar for OGM and SNP array, although there was evidence of genetic drift (Table 4). The ploidy for SK-N-BE(2)C was unclear as the SNP array BAF tracks suggest possible tetraploidy (Figure 4D).

#### 3.2.5. NBLW Cell Line

Our previous karyotyping (not shown) was consistent with the one reported by Foley [43]. The karyotype suggests large HSRs on both copies of chromosome 19 and the presence of extra material on chromosome 16 derived from chromosome 17.

There are 18 inter-chromosomal translocations and 18 intra-chromosomal fusions occurring within this cell line (Supplementary Table S5). *MYCN* is involved in 17 of the 18 translocations and all 18 intra-chromosomal fusions. This suggests hyper-rearrangement occurring on chromosome 2. Chromoplexy is likely to be due to the four-way translocation between chromosome 2 and chromosomes 1, 6 and 19 (Supplementary Figure S1A). Most translocations occurred between chromosomes 2 and 19, suggesting chromoplexy is occurring between these two chromosomes (Supplementary Figure S1B).

An amplicon is seen on chromosome 2 at the p24.3 locus (Supplementary Figure S1C,D). Here, the copy number is estimated at 175.33–415.52. Genes amplified in this amplicon are *NBAS* (oncogene), *DDX1* (protooncogene), *AK093525*, *MYCNOS* and *SNORA40*. *MYCN* has an estimated copy number of 278.47, *NBAS* of 200–400 and *DDX1* of 377.04. Within this locus are many structural variations, including inversions, deletions, translocations and duplications. There are also copy number increases on the q-arms of chromosomes 1 and 17, consistent with the SNP array (Supplementary Figure S1C,D).

### 3.3. Tumour Samples

#### 3.3.1. Tumour 1

**Figure 5:**
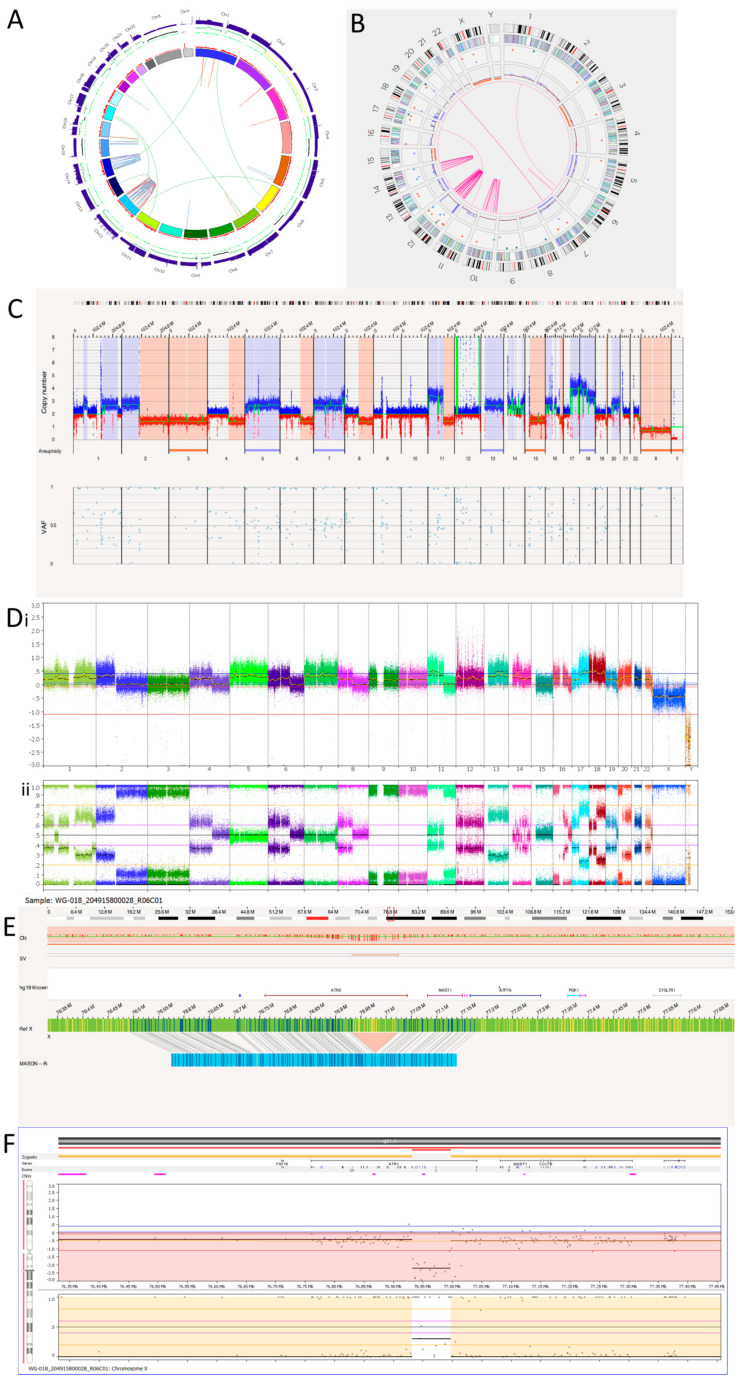
Neuroblastoma Tumour 1. (**A**) WGS Circos plot view. (**B**) OGM Circos plot view. (**C**) OGM whole genome view. OGM SV/SNV Key: Green, insertion; Orange, deletion; Light blue, inversion; Purple, duplication; Pink, Intra-chromosomal fusion or Inter-chromosomal Translocation; Yellow, LOH region; Dark blue, CNV Gain; Red, CNV Loss. (**D**) SNP array analysed using Nexus software. (**i**) log2 ratio. (**ii**) B allele frequency. (**E**) OGM view of intragenic *ATRX* deletion. The deletion is 91,132 bp long and located at Xq21.1. The red box represents the loci where the SV is present. (**F**) SNP array showing intragenic deletion of *ATRX*.

Tumour 1 was a metastatic relapse sample from a patient with non-*MYCN* amplified, high risk, NB (Table 2). The WGS Circos plot (Figure 5A) and SNP array (Figure 5D) show >10 oscillating copy numbers on chromosomes 12 and 14, suggesting chromothripsis is occurring on these chromosomes. This was confirmed by the OGM Circos plot due to the number of intra-chromosomal fusions occurring on these chromosomes (Figure 5B). OGM also identified chromothripsis occurring on chromosome 10. This was not detected by the SNP array or WGS as there were no copy number changes on chromosome 10. This may be due to spatial heterogeneity as a separate tumour sample was used for OGM, whereas the same DNA was used for the SNP array and WGS.

OGM detected 50 intra-chromosomal fusions in this tumour sample. Among these, 1 is located on chromosome 6, 11 on chromosome 10, 29 on chromosome 12 and 10 on chromosome 14. Table 8 shows overlapping genes involved in the intra-chromosomal fusions on the chromothriptic chromosomes. WGS was unable to detect the intra-chromosomal-fusion occurring on 6q that OGM identified. However, OGM was unable to detect the rearrangements occurring on the q-arm of chromosome 15 and translocations between chromosomes 15 and 16 called by WGS (Figure 5A and Table 9). In view of the low precision of the structural variant calling from short-read WGS using Manta [53], many of these calls may be false positives. 

All four inter-chromosomal translocations detected by OGM were also detected by WGS (Table 9). WGS also called an additional six translocations using Manta.

There is an amplicon at the 12p13.31 locus (Figure 5C,D) where the copy number is reported to be 10–15. Within the amplicon are seven deletions. Furthermore, there is a small amplicon located on chromosome 12q13.3. Within this amplicon is the *CDK4* gene, which has a copy number reported at around 10. Other genes located within this amplicon are *OS9*, *AGAP2*, *TSPAN31*, *DM110804*, *MARCH9*, *CYP27B1*, *METTL1*, *METTL21B*, *TSFM*, *AVIL*, *JAG611266*, *CTDSP2*, *MIR26A2*, *AK130110*, *LOC100506844* and *XRCC6BP1*.

Tumour 1 was found to have a deletion at the chromosome Xq21.1 locus, which was 91,312bp long and overlapped with *ATRX* (Figure 5E). This was an intragenic *ATRX* deletion which is associated with the presence of alternative lengthening of telomeres. Consistent with OGM findings, SNP array also detected an intragenic deletion involving exons 2 to 10 of *ATRX* (Figure 5F).

#### 3.3.2. Tumour 2

Tumour 2 was an intermediate-risk tumour taken from a patient post chemotherapy (Table 2). There were no translocations or fusions detected by OGM (Supplementary Figure S2B). Although the WGS Circos plot (Supplementary Figure S2A) shows four translocations, only one was included in the final report by Manta. This was mapped to t(9;11)(q33.2;q22.3). All copy number changes were whole chromosomal aberrations (Table 4, Supplementary Figure S2C,D). WGS estimated this to be a near-triploid tumour, which was inconsistent with the SNP array and FISH data (Supplementary Figure S2D), which called it a near-tetraploid tumour. OGM was unable to call ploidy accurately.

## 4. Discussion

In our study, five NB cell lines and two NB tumours were analysed using OGM on the Bionano Saphyr machine. Although not all the MQR value metrics were met for one of the cell lines (SH-SY5Y) and one of the tumour samples (Tumour 1), all samples were able to be analysed. For the SH-SY5Y cell line, the RNA fusion panel validated the fusion transcript detected by OGM, confirming reliability. OGM allows us to accurately view copy number changes, SVs and genomic rearrangements in one analysis, removing the extended time taken for additional tests and transfer of information between departments. Although sometimes technically difficult, it may prove useful in cancer cases with diagnostic difficulty, especially for detection of balanced translocations.

OGM uses a three-day protocol, with an additional two days for running the chip and pipeline analysis. It may be useful for patient samples in the diagnostic setting where turnaround time is increasingly important. SNP arrays take around one week to return results, and NHS England whole genome sequencing (WGS) currently takes at least 6–8 weeks to return results. However, if the DNA extraction is not completed correctly, it can cause the quality metrics to be below the recommended values and hinder further analysis. In addition, Bionano reagents and materials are expensive, although the cost of OGM may be less than the combination of multiple genetic tests. We have shown that a limitation of OGM is accurate ploidy detection and, therefore, FISH or SNP array may still be needed. In contrast, our preliminary data on two NB tumours suggest that it may be superior to WGS for accurate SV detection. OGM may also be comparable to SNP arrays for detecting CNAs, provided data quality is good, as our pilot analysis suggests. However, the lack of BAF data for OGM, especially for the rare variant analysis pipeline, is likely to result in a lower sensitivity compared to that of the SNP array. CNA evaluation will require a systematic comparison with larger sample sizes.

Genomic amplification is a frequent event in cancer, increasing gene copy number and resulting in increased activation and expression of proto-oncogenes, thus driving malignant transformation [21]. Amplifications are either extra-chromosomal DNA elements presenting as double minutes (DMs) or intra-chromosomal DNA elements presenting as HSRs [17]. These abnormalities have been seen in both NB cell lines and tumours and are frequently demonstrated through FISH at the site of amplified copies of *MYCN* [54]. *MYCN* amplification in NB presents as double minutes (DMs) in 93% of tumour cases, HSRs in 6%, and DMs and HSRs together in <1% [55]. In NB cell lines, HSRs occur more frequently, as shown by all three oncogene-amplified NB cell lines s in the current study harbouring HSRs. Previous studies have shown that DMs can integrate into host chromosomes, contributing to HSR evolution [56]. 

An emerging driver of amplification in a range of different cancers is seismic amplification. This is initiated by chromothripsis, followed by repetitive rounds of circular recombination of DNA fragments, leading to the final states of DMs and HSRs [17]. This has previously been reported in the NB cell line LS, where the amplicon with *MYCN* and *MDM2* was originally found to be circular extrachromosomal DNA that transformed into neochromosomes in later passages [17]. Amplicons found in cell lines and tumours in the current study may be a result of chromothripsis or chromoplexy. Furthermore, previous longitudinal analyses have shown that DMs can undergo continuous structural evolution to promote chromothriptic events by binding near chromosome ends and re-integrating when DNA damage occurs, contributing to increased drug tolerance [56]. DNA damage repair inhibitors may therefore be effective in cancers associated with chromothripsis such as NB. 

Another mechanism of focal oncogene amplification recently identified in breast cancer is through inter-chromosomal translocations secondary to oestrogen-induced DNA breaks [21]. This is termed translocation-bridge amplification, which involves repair through inter-chromosomal translocations and initiates a series of events leading to oncogene amplification [21]. Similar principles may apply where multiple breaks and repairs are occurring in chromothripsis [21]. This consequently leads to amplification similar to translocation-bridge amplification [21]. Whether oncogene amplification from such complex rearrangements and translocation-bridge amplification share mechanistic similarities in NB remains an avenue for further research. 

In the NB1691 cell line, an RNA fusion panel confirmed the presence of a fusion transcript between *BEST3* and *PTPRR*. *BEST3* encodes a protein and belongs to the bestrophin family [57,58], comprising three well-defined members (bestrophin 1–3) [58]. BEST3 is a major isoform of the family expressed in the cardiovascular system [58]. It has been hypothesised that a member of the bestrophin family is responsible for the cGMP-dependent Ca^2+^-activated Cl^−^ current in smooth muscles in the vascular wall [59]. The downregulation of *BEST3* mRNA and protein is associated with a significant reduction in the Cl^−^ current, suggesting BEST3 is essential for this process [59]. The protein encoded by *PTPRR* is a member of the protein tyrosine phosphatase (PTP) family, which are signalling molecules involved in regulating various cellular processes, including cell growth, differentiation, mitosis and oncogenic transformation [60]. PTPRR inhibits MAPK signalling by sequestering MAPKs in the cytoplasm in an inactive form [60,61]. In some cancers, such as breast and colon cancer, overexpression of a protein that silences *PTPRR* leads to activation of MAPK signalling and epithelial-mesenchymal transition (EMT) [61]. In the NB1691 cell line, PTPRR function is predicted to be disrupted by deletion of the phosphatase component upon formation of a fusion protein with BEST3, leading to activation of MAPK signalling and EMT. If confirmed, this could represent a novel mechanism of MAPK pathway activation in NB.

SH-SY5Y cells had a *PVT1::EXOC4* fusion gene detected by OGM, giving rise to a fusion transcript which was confirmed by the Illumina TruSight RNA fusion panel. EXOC4, exocyst complex component 4 gene, is involved in vesicular transport and has a super-enhancer within its locus, which, in SH-SY5Y cells, has been reported to be translocated to less than a megabase downstream from the *C-MYC* transcriptional start site [48]. As a result, it leads to increased *C-MYC* expression through enhancer hijacking. *PVT1* is in the same locus as *MYC* (55 kb away), 8q24.21. It is a long non-coding RNA (lncRNA) which displays oncogenic functions in several different cancer types [49] and is a strong activator of cellular proliferation and tumour growth [47,49]. *PVT1* fusion transcripts with *MYC* have been identified in solid cancers which typically arise from translocations within this locus [49]. The *PVT1* gene contains two non-canonical *MYC*-binding sites in the promoter region, and multiple studies have established that there are regulatory networks between *PVT1* and *MYC* [47]. One example of this is in medulloblastoma, where *MYC* was shown to increase its own expression by binding *PVT1* in a positive feedback loop [47]. Our study has extended previous findings by suggesting that in SH-SY5Y cells, *PVT1* also has a role in MYC regulation through the non-canonical *MYC*-binding sites, and together with the *EXOC4* enhancer-hijacking properties, they account for the high *MYC* expression reported in this cell line.

OGM is able to detect TMMs in both NB cell lines and tumours. It can reliably identify *MYCN* amplification, *TERT* rearrangements and *ATRX* deletions using a single technique. Although unable to measure the length of telomeres using the T2T reference, OGM can detect other TMMs. In this study, OGM detected *MYCN* amplification in three cell lines (NB1691, NBLW and SK-N-BE2(C)), a *TERT* rearrangement in one cell line (GI-ME-N) and an intragenic *ATRX* deletion in a NB tumour. The clinical significance of TMMs in NB is becoming increasingly clear, and the ability to detect TMMs using one technique raises the possibility that OGM could become a critical test for NB molecular diagnostics in the future.

## 5. Conclusions

The ability of OGM to combine multiple genetic analyses, reduce overall cost and turnaround time and improve diagnostic accuracy by detecting novel SVs supports its adoption to frontline clinical genetic testing in the future. The detection of translocations, amplifications and chromosomal damage all in one test is currently not yet possible in NHS diagnostic labs. OGM allows for the interrogation of complex genomes and identification of genetic alterations which may play a role in the development and prognosis of NB and other cancers. 

Here, we show the utility of OGM to refine SV detection and predict underlying mechanisms of gene amplification in NB, including the detection of novel SVs of potential clinical significance. We conclude that it is a useful adjunct to standard genetic analyses of NB and should be further evaluated in a larger comparative study in the future.

## Figures and Tables

**Table 1:** Characteristics of NB cell lines. M = male; F = female; TMM = telomere maintenance mechanisms; *TERT* = telomere reverse transcriptase; amp = amplified; non-amp = non-amplified. WT = Wild-type. MNA = *MYCN* amplification.

Cell Line	Sex	Age	Origin	TMM Status	*MYCN* Amp	*TP53* Status	*ALK* Status	*MDM2* Amp	Reference
**NB1691**	M	1.9 y	Derived from a recurrent retroperitoneal NB tumour	MNA (*TERT* + ve)	Amp	WT	Amp	Amp	[35,36]
**SH-SY5Y**	F	4 y	Clone of the SKNSH cell line derived from a bone marrow biopsy from a relapsed metastatic NB	C288T *TERT* promoter mutation (*TERT* + ve)	Non-amp	WT	Mutant F1174L	Non-amp	[37,38]
**GI-ME-N**	F	2 y	Derived from bone marrow of a patient with stage 4 NB after six months of chemotherapy	*TERT* rearrangement (*TERT* + ve)	Non-amp	WT	WT	Non-amp	[10,39]
**SK-N-BE(2)C**	M	22 mo	Subclone of the SK-N-BE(2) NB cell line taken from bone marrow after chemotherapy and radiotherapy	MNA (*TERT* + ve)	Amp	Mutant C135F	WT	Non-amp	[40,41]
**NBLW**	M	6 mo	Derived from the primary adrenal tumour of a child with pre-treatment metastatic stage 4S NB with metastasis to the liver	MNA (*TERT* + ve)	amp	WT	Mutant R1275L	Non-amp	[42,43]

**Table 2:** Clinical information for NB tumour samples. M = male; R = relapse; PC = post-chemotherapy; Int = intermediate; INRG = international neuroblastoma risk group; INSS = international neuroblastoma staging system; M = metastatic; L2 = localised unresectable; UH = unfavourable histology; MNA = *MYCN* amplified; DOD = died of disease; ADF = alive disease free; TCC = tumour cell content.

Patient ID	Sex	Age at Diagnosis	Sample	INRG Risk	INSS	INRG Stage	Histology	*MYCN* Status	Status	TCC%
**1**	M	5 y, 2 mo	R	High	4	M	UH	Non-MNA	DOD	90
**2**	M	2 y, 2 mo	PC	Int	3	L2	UH	Non-MNA	ADF	60

**Table 3:** Molecule quality report (MQR) for the five neuroblastoma cell lines and two neuroblastoma tumour samples, including recommended values and definitions. Kbp = kilobase pairs; Gbp = giga base pairs; N50 = the sequence length of the shortest molecule at 50% total molecule length. * Effective coverage should be 400× for rare variant analysis and 80× for de novo analysis.

	Map Rate (%)	Label Density (/1000 kbp)	Effective Coverage	Molecules N50 (≥150 kbp and min Sites ≥ 9) (kbp)	Total DNA (≥150 kbp) (Gbp)
**SH-SY5Y**	56.0	12.33	276.20	215.27	1601.27
**NBLW**	78.3	13.87	391.47	329.92	1600.44
**NB1691**	61.3	10.57	94.32	242.25	582.80
**GI-ME-N**	77.6	15.74	357.87	308.63	1470.66
**SK-N-BE(2)C**	92.4	15.50	469.56	310.88	1627.05
**Tumour 1**	63.1	19.32	317.46	279.00	1610.73
**Tumour 2**	82.4	15.40	410.41	253.13	1600.89
**Recommended Values**	*≥70*	*14–17*	~400x/~80x *	*≥230*	*~1600*
**Definitions**	*Percentage of molecules that are 150 kbp or longer and mapped to* *the hg19 genome reference*	*Average number of labels per 100 kbp for the molecules that are 150 kbp or longer*	*Total amount of aligned DNA divided by the size of the reference genome times the map rate*	*N50 of the molecules that are 150 kbp or longer, but molecules must have at least 9 labels*	*Total amount of DNA from molecules that are 150 kbp or longer*

**Table 4:** Comparison of CNAs found using OGM and SNP array. CNA calls in bold were uniquely detected using the specific method. Chromosomes reported to have complex CNAs include chromothripsis or hyper-rearrangement.

		Ploidy	SNP Array	OGM
Cell Line	NB1691	2n	+7, +17, +22; +1p, +1q, +5p, +6p, +6q, +12p, +12q, +16q, +19p, +20q, +20q, +Xq Amplicons: 2p, 12q Complex: Chr5	+7, +17, +22; +1p, +1q, +5p, +6p, +6q, +12p, +12q, +16q, +19p, +20q, +20q, +Xq Amplicons: 2p, 12q Complex: Chr5
	SH-SY5Y	2n	+7; +1q, +17q; −14p, −22q	+7, **−19**; +1q, +17q; **−1p**, **−4p**, **−9q**, −14q, −22q **Complex: Chr7**
	GI-ME-N	4n	−7, −9, −13, −15, −20, −21, −22; +4q, +11q, +17q, +19q, +Xp, +Xq; −1p, −2p, −2q, −4q, −4q, −5p, −5q, −6p, −6q, −6q, −10pq, −11p, −11q, −18p, −18p, −18q	−7, −9, −13, −15, −20, −21, −22; +4q, +11q, +17q, +19q, +Xp, +Xq; −1p, −2p, −2q, −4q, −4q, −5p, −5q, −6p, −6q, −6q, −10pq, −11p, −11q, −18p, −18p, −18q
	SK-N-BE(2)C	2n/4n	**−4**, **−5**, **−10**, **−13**, −18, **−21**; −1p, −2q, −3p, **−6p**, −9p, −9p, −11p, **−16q**, −17p, −20p, **−Xq**; Amplicon: 2p **Complex: Chr4**	−18; −1p, −2q, −3p, −9p, −11p, −17p, −20p Amplicon: 2p Complex:—
	NBLW	2n	+1q, +2p, +17q Amplicon: 2p	+1q, +2p, +17q Amplicon: 2p
Tumour	T1	3n	+5, +13; −3, −15; +1p, +1q, +2p, +7p, +7q, +7q, +7q, +11p, +16p, +16q, +17q, +18p, +18q, +20q; −2q, −4q, −6q, −8q, −11q, −16q, −19q, −22q Amplicons: Chr12 Complex: Chr12, chr14 *ATRX* deletion	+5, +13; −3, −15; +1p, +1q, +2p, +7p, +7q, +7q, +7q, +11p, +16p, +16q, +17q, +18p, +18q, +20q; −2q, −4q, −6q, −8q, −11q, −16q, −19q, −22q Amplicons: Chr12 Complex: Chr12, chr14 *ATRX* deletion
	T2	4n	−3, −4, −14, −15, −16	**+6**, **+7**, **+9**, **+11**, **+17**; −3, −4, −14, −15, −16

**Table 5:** Translocations and intra-chromosomal fusions present in the SH-SY5Y cell line detected using OGM. Confidence should be >0.05. Self-molecule count should be ≥5.

	Location	Overlap Gene(s)	Confidence	Self-Molecule Count
**Inter -chromosomal Translocations**	t(17;22)(q21.31;q13.1)	*MICALL1*	0.91	17
	t(7;8)(q33;q24.21)	*EXOC4;PVT1*	0.61	36
	t(7;8)(q33;q24.21)	*EXOC4;PVT1*	0.82	23
	t(7;8)(q33;q24.21)	*EXOC4;PVT1*	0.70	26
	t(15;22)(q24.2;q12.3)	*NPTN;SLC5A1*	0.07	16
	t(15;22)(q24.2;q12.3)	*NPTN;SLC5A1*	0.24	16
	t(15;22)(q24.2;q12.3)		0.10	20
	t(15;22)(q24.2;q12.3)		0.52	22
**Intra-chromosomal Fusions**	inv(4)(p15.33p15.1)		0.16	7
	inv(4)(p15.33p15.1)		0.38	7
	inv(1)(q21.1q32.1)		0.06	65
	inv(14)(q13.3q21.3)	*PAX9*	0.15	30

**Table 6:** Translocations and intra-chromosomal fusions present in the GI-ME-N cell line detected using OGM. Confidence should be >0.05 and self-molecule count should be ≥5 for a translocation/intra-chromosomal fusion to be reliable. * Nearest non-overlap gene.

	Location	Overlap Gene(s)	Confidence	Self-Molecule Count
Translocations	t(4;5)(p15.1;p13.3)		0.08	16
	t(5;19)(p15.33;q13.43)	TERT *	0.12	25
	t(1;17)(p35.2;q11.2)	*NF1*	0.08	19
	t(1;17)(p35.2;q11.2)	*NF1*	0.1	18
	t(11;17)(q13.4;q12)	*SHANK2;AP2B1*	0.13	33
	t(16;19)(q24.3;q13.33)	*MYH14*	0.07	24
	t(16;19)(q24.3;q13.33)	*MYH14*	0.31	26
	t(6;19)(p21.31;q132.2)	*ZNF574*	0.71	11
	t(6;19)(p21.31;q132.2)	*ZNF574*	0.80	14
	t(6;19)(p21.31;q132.2)	*FKBP5*	0.24	17
	t(6;19)(p21.31;q132.2)	*FKBP5*	0.56	16
	t(4;11)(q34.2;p11.2)	*SPCS3*	0.32	21
	t(6;10)(q14.1;p14)		0.66	26
Intrachromosomal-Fusions	inv(18)(p11.32p11.31)	*COLEC12*	0.06	27
	inv(4)(q25q33)		0.23	8
	inv(2)(p23.3p16.1)	*RAB10;CCDC85A*	0.38	10

**Table 7:** Translocations and intra-chromosomal fusions present in the SK-N-BE(2)C cell line detected using OGM. Confidence should be >0.05 and self-molecule count should be ≥5 for a translocation/intra-chromosomal fusion to be reliably called.

	Location	Overlap Gene(s)	Confidence	Self-Molecule Count
**Interchromosomal Translocations**	t(1;2)(p21.3;p16.3)		0.03	30
	t(1;2)(p21.3;p16.3)		0.02	30
	t(16;19)(p11.2;p13.3)	*SRCAP;GNG7*	0.47	24
	t(16;19)(p11.2;p13.3)	*SRCAP;GNG7*	0.13	23
	t(16;19)(p11.2;p13.3)	*SRCAP;GNG7*	0.32	11
	t(16;19)(p11.2;p13.3)	*SRCAP;GNG7*	0.20	10
	t(2;6)(p24.3;p21.1)	*CDC5L*	0.63	24
	t(2;6)(p24.3;p21.1)	*CDC5L*	0.26	17
	t(2;4)(p24.3;q22.3)		0.56	13
	t(2;4)(p24.3;q22.3)	*MYCN;SMARCAD1*	0.71	18
	t(11;20)(q13.1;p15)		0.36	21
	t(11;20)(q13.1;p15)		0.07	18
	t(3;17)(p14.2;q11.2)		0.71	25
**Intrachromosomal Fusions**	inv(2)(p24.3)	*MYCN*	0.90	2277
	inv(2)(p24.3)	*MYCN*	0.87	1691
	inv(2)(p24.3)	*MYCN*	0.83	90
	inv(2)(p24.3)	*MYCN*	0.71	1869
	inv(2)(p24.3)	*MYCN*	0.60	2064
	inv(2)(p24.3)	*MYCN*	0.44	1407
	inv(2)(p24.3)	*MYCN*	0.38	1940
	inv(2)(p24.3)	*MYCN*	0.34	1697
	inv(2)(p24.3)	*MYCN*	0.31	2131
	inv(2)(p24.3)	*MYCN*	0.28	1299
	inv(2)(p24.3)	*MYCN*	0.28	972
	inv(2)(p24.3)	*MYCN*	0.08	1174

**Table 8:** Overlap genes involved in intra-chromosomal fusions on chromosomes with chromothripsis detected using OGM in Tumour 1.

Chromosome	Overlap Genes
10	*FBXW4, ZMIZ1, PFKP, STAM, NT5C2, A1CF, GBF1, PAX2*
12	*SYNE2, AKAPB, C12orf, ESRRB, JDP2, VIPAS39, UNC79, TRAF3*
14	*CLEC12A, CCDC64, NAV3, PTPRO, OS9, XRCC6BP1, SLC6A12, FGD6, NELL2, SRRM4, FAM19A2, APAF1, ZDHHC17, ANO4*

**Table 9:** Translocations and hyper-rearrangements in the Tumour 1 sample detected by OGM and WGS. Chr = chromosome.

Translocation	OGM Coordinates (GRCh37)	WGS Coordinates (GRCh38)	Match
t(1;6)(q21.3;q16.3)	chr1:150,853,601; chr6:104,007,705	1:150871900; ]6:103570849]C	Yes
t(1;14)(p31.1;q12)	-	1:71560430; T]14:27860966]	No
t(6;12)(p25.2;p13.33)	-	6:3021510; T [12:1788929[	No
t(7;20)(q21.12;q13.33)	chr7: 86,811,692; chr20:62,427,892	7:87177999; ]20:63799630]A	Yes
t(7;20)(q21.12;q13.33)	chr7: 87,695,509; chr20:62,388,312	7:88067384; T [20:63755363[	Yes
t(11;17)(q14.1;q12)	chr11: 78,017,590; chr17:34,015,764	11:78308141; T [17:35687851[	Yes
t(15;16)(q26.1;p13.3)	-	15:90362018; [16:2115042[C	No
t(15;16)(q26.1;p13.3)	-	15:90362040; [16:2103430[T	No
t(15;16)(q26.1;p13.3)	-	15:90362495; G]16:2103629]	No
t(15;16)(q26.1;p13.3)	-	15:90593439; T [16:2115136[	No
**Hyper-rearrangement**	**OGM present**	**WGS present**	**Match**
Chr10 hyper-rearrangement	Present	Not detected	No
Chr12 hyper-rearrangement	Present	Present	Yes
Chr14 hyper-rearrangement	Present	Present	Yes

## Data Availability

The data shown in this study are available in this manuscript and the supplementary material.

## Supplementary Materials

The following supporting information can be downloaded at: https://www.mdpi.com/article/10.3390/cancers15215233/s1. The following supporting information can be found in an additional document submitted: Table S1: Table to show 507 identifiable genes found on the RNA fusion panel; Table S2: Filter settings according to the recommendations of Bionano; Table S3: Translocations and intra-chromosomal fusions in the NB1691 neuroblastoma cell line; Table S4: RNA fusion panel results; Table S5: Translocations and intra-chromosomal fusions in the NBLW neuroblastoma cell line; 2; Figure S1: NBLW neuroblastoma cell line; Figure S2: Neuroblastoma Tumour 2.

## Abbreviations

SV, structural variant; OGM, optical genome mapping; LOH, loss of heterozygosity.

## References

[1] Zafar A., Wang W., Liu G., Wang X., Xian W., McKeon F., Foster J., Zhou J., Zhang R. (2021). Molecular targeting therapies for neuroblastoma: Progress and challenges. Med. Res. Rev..

[2] Matthay K.K., Maris J.M., Schleiermacher G., Nakagawara A., Mackall C.L., Diller L., Weiss W.A. (2016). Neuroblastoma. Nat. Rev. Dis. Prim..

[3] Kimura S., Sekiguchi M., Watanabe K., Hiwatarai M., Seki M., Yoshida K., Isobe T., Shiozawa Y., Suzuki H., Hoshino N. (2021). Association of high-risk neuroblastoma classification based on expression profiles with differentiation and metabolism. PLoS ONE.

[4] Brodeur G.M. (2003). Neuroblastoma: Biological insights into a clinical enigma. Nat. Rev. Cancer.

[5] Cohn S.L., Pearson A.D., London W.B., Monclair T., Ambros P.F., Brodeur G.M., Faldum A., Hero B., Iehara T., Machin D. (2009). The International Neuroblastoma Risk Group (INRG) classification system: An INRG Task Force report. J. Clin. Oncol..

[6] Brodeur G.M., Pritchard J., Berthold F., Carlsen N.L., Castel V., Castelberry R.P., De Bernardi B., Evans A.E., Favrot M., Hedborg F. (1993). Revisions of the international criteria for neuroblastoma diagnosis, staging, and response to treatment. J. Clin. Oncol..

[7] Cohn S.L., Tweddle D.A. (2004). MYCN amplification remains prognostically strong 20 years after its “clinical debut”. Eur. J. Cancer.

[8] Molenaar J.J., Koster J., Zwijnenburg D.A., van Sluis P., Valentijn L.J., van der Ploeg I., Hamdi M., van Nes J., Westerman B.A., van Arkel J. (2012). Sequencing of neuroblastoma identifies chromothripsis and defects in neuritogenesis genes. Nature.

[9] Schleiermacher G., Javanmardi N., Bernard V., Leroy Q., Cappo J., Rio Frio T., Pierron G., Lapouble E., Combaret V., Speleman F. (2014). Emergence of new ALK mutations at relapse of neuroblastoma. J. Clin. Oncol..

[10] Peifer M., Hertwig F., Roels F., Dreidax D., Gartlgruber M., Menon R., Krämer A., Roncaioli J.L., Sand F., Heuckmann J.M. (2015). Telomerase activation by genomic rearrangements in high-risk neuroblastoma. Nature.

[11] Allinson L.M., Potts A., Goodman A., Bown N., Bashton M., Thompson D., Basta N.O., Gabriel A.S., McCorkindale M., Ng A. (2022). Loss of ALK hotspot mutations in relapsed neuroblastoma. Genes Chromosom. Cancer.

[12] Eleveld T.F., Oldridge D.A., Bernard V., Koster J., Colmet Daage L., Diskin S.J., Schild L., Bentahar N.B., Bellini A., Chicard M. (2015). Relapsed neuroblastomas show frequent RAS-MAPK pathway mutations. Nat. Genet..

[13] Schleiermacher G., Michon J., Ribeiro A., Pierron G., Mosseri V., Rubie H., Munzer C., Bénard J., Auger N., Combaret V. (2011). Segmental chromosomal alterations lead to a higher risk of relapse in infants with MYCN-non-amplified localised unresectable/disseminated neuroblastoma (a SIOPEN collaborative study). Br. J. Cancer.

[14] Janoueix-Lerosey I., Schleiermacher G., Michels E., Mosseri V., Ribeiro A., Lequin D., Vermeulen J., Couturier J., Peuchmaur M., Valent A. (2009). Overall genomic pattern is a predictor of outcome in neuroblastoma. J. Clin. Oncol..

[15] Pellestor F. (2019). Chromoanagenesis: Cataclysms behind complex chromosomal rearrangements. Mol. Cytogenet..

[16] Stephens P.J., Greenman C.D., Fu B., Yang F., Bignell G.R., Mudie L.J., Pleasance E.D., Lau K.W., Beare D., Stebbings L.A. (2011). Massive genomic rearrangement acquired in a single catastrophic event during cancer development. Cell.

[17] Rosswog C., Bartenhagen C., Welte A., Kahlert Y., Hemstedt N., Lorenz W., Cartolano M., Ackermann S., Perner S., Vogel W. (2021). Chromothripsis followed by circular recombination drives oncogene amplification in human cancer. Nat. Genet..

[18] Cortés-Ciriano I., Gulhan D.C., Lee J.J., Melloni G.E.M., Park P.J. (2022). Computational analysis of cancer genome sequencing data. Nat. Rev. Genet..

[19] Shen M.M. (2013). Chromoplexy: A new category of complex rearrangements in the cancer genome. Cancer Cell.

[20] Baca S.C., Prandi D., Lawrence M.S., Mosquera J.M., Romanel A., Drier Y., Park K., Kitabayashi N., MacDonald T.Y., Ghandi M. (2013). Punctuated evolution of prostate cancer genomes. Cell.

[21] Lee J.J., Jung Y.L., Cheong T.C., Espejo Valle-Inclan J., Chu C., Gulhan D.C., Ljungström V., Jin H., Viswanadham V.V., Watson E.V. (2023). ERα-associated translocations underlie oncogene amplifications in breast cancer. Nature.

[22] Lundberg G., Sehic D., Länsberg J.K., Øra I., Frigyesi A., Castel V., Navarro S., Piqueras M., Martinsson T., Noguera R. (2011). Alternative lengthening of telomeres—An enhanced chromosomal instability in aggressive non-MYCN amplified and telomere elongated neuroblastomas. Genes Chromosom. Cancer.

[23] Shay J.W., Wright W.E. (2000). Hayflick, his limit, and cellular ageing. Nat. Rev. Mol. Cell Biol..

[24] Dratwa M., Wysoczańska B., Łacina P., Kubik T., Bogunia-Kubik K. (2020). TERT-Regulation and Roles in Cancer Formation. Front. Immunol..

[25] Nabetani A., Ishikawa F. (2011). Alternative lengthening of telomeres pathway: Recombination-mediated telomere maintenance mechanism in human cells. J. Biochem..

[26] Farooqi A.S., Dagg R.A., Choi L.M., Shay J.W., Reynolds C.P., Lau L.M. (2014). Alternative lengthening of telomeres in neuroblastoma cell lines is associated with a lack of MYCN genomic amplification and with p53 pathway aberrations. J. Neurooncol..

[27] Mantere T., Neveling K., Pebrel-Richard C., Benoist M., van der Zande G., Kater-Baats E., Baatout I., van Beek R., Yammine T., Oorsprong M. (2021). Optical genome mapping enables constitutional chromosomal aberration detection. Am. J. Hum. Genet..

[28] Biomnis Constitutional Cyto- and Molecular Genetics: Karyotyping, FISH and CGH Array. https://www.eurofins-biomnis.com/wp-content/uploads/2016/04/56-INTGB-Focus_Karyotyping_SNP_array.pdf.

[29] BionanoGenomics Revolutionising Cytogenomics. https://bionanogenomics.com/wp-content/uploads/2022/05/30368_Rev.C_Cytogenetics-Vignette_DIGITAL.pdf.

[30] Genomics B. See Structural Variation Like Never before with Bionano Optical Genome Mapping. https://eira.ams3.cdn.digitaloceanspaces.com/files/referrals/bOHnz1U922QRjdNU4veKMJga4Jz4JZKV55HB6Gp9.pdf.

[31] BionanoGenomics Hematological Malignancies and Solid Tumour Research. https://bionanogenomics.com/wp-content/uploads/2022/05/30367_-Rev.B-_Cancer-Vignettes-Effective_DIGITAL.pdf.

[32] Hoyt S.J., Storer J.M., Hartley G.A., Grady P.G.S., Gershman A., de Lima L.G., Limouse C., Halabian R., Wojenski L., Rodriguez M. (2022). From telomere to telomere: The transcriptional and epigenetic state of human repeat elements. Science.

[33] Tembrink M., Gerding W.M., Wieczorek S., Mika T., Schroers R., Nguyen H.P., Vangala D.B., Nilius-Eliliwi V. (2023). Novel NUP98::ASH1L Gene Fusion in Acute Myeloid Leukemia Detected by Optical Genome Mapping. Cancers.

[34] Peng Y., Yuan C., Tao X., Zhao Y., Yao X., Zhuge L., Huang J., Zheng Q., Zhang Y., Hong H. (2020). Integrated analysis of optical mapping and whole-genome sequencing reveals intratumoral genetic heterogeneity in metastatic lung squamous cell carcinoma. Transl. Lung Cancer Res..

[35] Wong K.E., Mora M.C., Sultana N., Moriarty K.P., Arenas R.B., Yadava N., Schneider S.S., Tirabassi M.V. (2018). Evaluation of Rhodiola crenulata on growth and metabolism of NB-1691, an MYCN-amplified neuroblastoma cell line. Tumour Biol..

[36] Tucker E.R., Jiménez I., Chen L., Bellini A., Gorrini C., Calton E., Gao Q., Che H., Poon E., Jamin Y. (2023). Combination Therapies Targeting ALK-aberrant Neuroblastoma in Preclinical Models. Clin. Cancer Res..

[37] Lindner S., Bachmann H.S., Odersky A., Schaefers S., Klein-Hitpass L., Hero B., Fischer M., Eggert A., Schramm A., Schulte J.H. (2015). Absence of telomerase reverse transcriptase promoter mutations in neuroblastoma. Biomed. Rep..

[38] Biedler J.L., Roffler-Tarlov S., Schachner M., Freedman L.S. (1978). Multiple neurotransmitter synthesis by human neuroblastoma cell lines and clones. Cancer Res..

[39] Cornaglia-Ferraris P., Ponzoni M., Montaldo P., Mariottini G.L., Donti E., Di Martino D., Tonini G.P. (1990). A new human highly tumorigenic neuroblastoma cell line with undetectable expression of N-myc. Pediatr. Res..

[40] Tweddle D.A., Malcolm A.J., Bown N., Pearson A.D., Lunec J. (2001). Evidence for the development of p53 mutations after cytotoxic therapy in a neuroblastoma cell line. Cancer Res..

[41] Biedler J.L., Spengler B.A., Ross R.A. (1979). Chromosomal and biochemical properties of human neuroblastoma cell lines and clones in cell culture. Gaslini.

[42] Chen L., Humphreys A., Turnbull L., Bellini A., Schleiermacher G., Salwen H., Cohn S.L., Bown N., Tweddle D.A. (2016). Identification of different ALK mutations in a pair of neuroblastoma cell lines established at diagnosis and relapse. Oncotarget.

[43] Foley J., Cohn S.L., Salwen H.R., Chagnovich D., Cowan J., Mason K.L., Parysek L.M. (1991). Differential expression of N-myc in phenotypically distinct subclones of a human neuroblastoma cell line. Cancer Res..

[44] QIAGEN AllPrep DNA/RNA Mini Handbook. www.qiagen.com/us/resources/resourcedetail?id=580866a6-56c6-4674-8566-2852164d8519&lang=en.

[45] Illumina Automatic Data Analysis. RNA Fusion Analysis Module. https://emea.illumina.com/products/by-type/informatics-products/local-run-manager.html.

[46] Depuydt P., Boeva V., Hocking T.D., Cannoodt R., Ambros I.M., Ambros P.F., Asgharzadeh S., Attiyeh E.F., Combaret V., Defferrari R. (2018). Genomic Amplifications and Distal 6q Loss: Novel Markers for Poor Survival in High-risk Neuroblastoma Patients. J. Natl. Cancer Inst..

[47] Onagoruwa O.T., Pal G., Ochu C., Ogunwobi O.O. (2020). Oncogenic Role of PVT1 and Therapeutic Implications. Front. Oncol..

[48] Zimmerman M.W., Liu Y., He S., Durbin A.D., Abraham B.J., Easton J., Shao Y., Xu B., Zhu S., Zhang X. (2018). MYC Drives a Subset of High-Risk Pediatric Neuroblastomas and Is Activated through Mechanisms Including Enhancer Hijacking and Focal Enhancer Amplification. Cancer Discov..

[49] Tolomeo D., Agostini A., Visci G., Traversa D., Storlazzi C.T. (2021). PVT1: A long non-coding RNA recurrently involved in neoplasia-associated fusion transcripts. Gene.

[50] TP73 Tumour Protein p73. https://www.ncbi.nlm.nih.gov/gtr/genes/7161/.

[51] Zeineldin M., Federico S., Chen X., Fan Y., Xu B., Stewart E., Zhou X., Jeon J., Griffiths L., Nguyen R. (2020). MYCN amplification and ATRX mutations are incompatible in neuroblastoma. Nat. Commun..

[52] Hölzel M., Huang S., Koster J., Ora I., Lakeman A., Caron H., Nijkamp W., Xie J., Callens T., Asgharzadeh S. (2010). NF1 is a tumor suppressor in neuroblastoma that determines retinoic acid response and disease outcome. Cell.

[53] Chen X., Schulz-Trieglaff O., Shaw R., Barnes B., Schlesinger F., Källberg M., Cox A.J., Kruglyak S., Saunders C.T. (2016). Manta: Rapid detection of structural variants and indels for germline and cancer sequencing applications. Bioinformatics.

[54] Emanuel B.S., Balaban G., Boyd J.P., Grossman A., Negishi M., Parmiter A., Glick M.C. (1985). N-myc amplification in multiple homogeneously staining regions in two human neuroblastomas. Proc. Natl. Acad. Sci. USA.

[55] Moreau L.A., McGrady P., London W.B., Shimada H., Cohn S.L., Maris J.M., Diller L., Look A.T., George R.E. (2006). Does MYCN amplification manifested as homogeneously staining regions at diagnosis predict a worse outcome in children with neuroblastoma? A Children’s Oncology Group study. Clin. Cancer Res..

[56] Shoshani O., Brunner S.F., Yaeger R., Ly P., Nechemia-Arbely Y., Kim D.H., Fang R., Castillon G.A., Yu M., Li J.S.Z. (2021). Chromothripsis drives the evolution of gene amplification in cancer. Nature.

[57] GeneCards BEST3 Gene—Bestrophin 3. https://www.genecards.org/cgi-bin/carddisp.pl?gene=BEST3#:~:text=GeneCards%20Summary%20for%20BEST3%20Gene,anions%20and%20amino%20acids%2Foligopeptides.

[58] Zhang T.T., Lei Q.Q., He J., Guan X., Zhang X., Huang Y., Zhou Z.Y., Fan R.X., Wang T., Li C.X. (2023). Bestrophin3 Deficiency in Vascular Smooth Muscle Cells Activates MEKK2/3-MAPK Signaling to Trigger Spontaneous Aortic Dissection. Circulation.

[59] Matchkov V.V., Larsen P., Bouzinova E.V., Rojek A., Boedtkjer D.M., Golubinskaya V., Pedersen F.S., Aalkjaer C., Nilsson H. (2008). Bestrophin-3 (vitelliform macular dystrophy 2-like 3 protein) is essential for the cGMP-dependent calcium-activated chloride conductance in vascular smooth muscle cells. Circ. Res..

[60] GeneCards PTPRR Gene—Protein Tyrosine Phosphatase Receptor Type R. https://maayanlab.cloud/Harmonizome/gene/PTPRR.

[61] Su P.H., Lin Y.W., Huang R.L., Liao Y.P., Lee H.Y., Wang H.C., Chao T.K., Chen C.K., Chan M.W., Chu T.Y. (2013). Epigenetic silencing of PTPRR activates MAPK signaling, promotes metastasis and serves as a biomarker of invasive cervical cancer. Oncogene.

