# Supplementary material for: Use of Optical Genome Mapping to Detect Structural Variants in Neuroblastoma
Source: Cancers (Basel). 2023 Oct 31;15(21):5233. doi: 10.3390/cancers15215233 (PMC10647738; doi:10.3390/cancers15215233)

Supplementary Material

Supplementary Table S1: Table to show the 507 identifiable genes found on the fusion panel

ABI1	*BRWD3*	*COL1A2*	*ERLIN2*	*HHEX*	*LNP1*	*NFIB*	*POU5F1*	*SLC34A2*	*TGFBR3*
ABL1	*BTBD18*	*COL6A3*	*ESR1*	*HIP1*	*LPP*	*NGF*	*PPAP2B*	*SLC45A3*	*THADA*
ABL2	*BTG1*	*COX6C*	*ETS1*	*HIPK1*	*LPXN*	*NGFR*	*PPARG*	*SLCO1B3*	*THRAP3*
ACACA	*C11orf1*	*CPDF6*	*ETV1*	*HIST1H4I*	*LRMP*	*NIN*	*PPARGC1A*	*SMAP1*	*TIRAP*
ACE	*C11orf95*	*CRADD*	*ETV4*	*HLF*	*LRRC37B*	*NIPBL*	*PPFIBP1*	*SMARCA5*	*TLX1*
ACER1	*C2CD2L*	*CREB1*	*ETV5*	*HMGA2*	*LTBP1*	*NKX2-5*	*PPP2R1B*	*SMARCB1*	*TLX3*
ACKR3	*C3orf27*	*CREB3L1*	*ETV6*	*HNF1A*	*LYL1*	*NONO*	*PRCC*	*SNHG5*	*TMPRSS2*
ACSL6	*CAMTA1*	*CREB3L2*	*EWSR1*	*HOXA10*	*MACROD1*	*NOTCH1*	*PRDM16*	*SORBS2*	*TNFRSF17*
ADD3	*CAPRIN1*	*CREBBP*	*EZR*	*HOXA11*	*MAF*	*NPM1*	*PRKACA*	*SORT1*	*TOP1*
AFF1	*CARS*	*CRLF2*	*FAM19A2*	*HOXA13*	*MAFB*	*NR4A3*	*PRKAR1A*	*SP3*	*TOP2B*
AFF3	*CASC5*	*CRTC1*	*FCGR2B*	*HOXA9*	*MALT1*	*NR6A1*	*PRKG2*	*SPECC1*	*TP53BP1*
AFF4	*CASP7*	*CSF1*	*FCRL4*	*HOXC11*	*MAML2*	*NSD1*	*PRRX2*	*SPTBN1*	*TPM3*
AGR3	*CBFA2T3*	*CSF1R*	*FEN1*	*HOXC13*	*MAPRE1*	*NT5C2*	*PSIP1*	*SQSTM1*	*TPM4*
AHI1	*CBFB*	*CTDSP2*	*FEV*	*HOXD11*	*MBNL1*	*NTF3*	*PSMD2*	*SRF*	*TRHDE*
AHRR	*CBL*	*CTNNB1*	*FGF8*	*HOXD13*	*MBTD1*	*NTF4*	*PTPRR*	*SRSF3*	*TRIM24*
ALK	*CCAR2*	*CUX1*	*FGFR1*	*HSP90AA1*	*MDS2*	*NTRK1*	*PVT1*	*SS18*	*TRIP11*
ANKRD28	*CCDC28A*	*DAB2IP*	*FGFR1OP*	*ID4*	*MEAF6*	*NTRK2*	*RABEP1*	*SS18L1*	*TRPS1*
AR	*CCDC6*	*DACH1*	*FGFR1OP2*	*IKZF1*	*MECOM*	*NTRK3*	*RAD51B*	*SSBP2*	*USP16*
ARHGAP20	*CCDC88C*	*DACH2*	*FGFR2*	*IL2*	*MGEA5*	*NUMA1*	*RAF1*	*SSX1*	*USP42*
ARHGAP26	*CCNB1IP1*	*DDIT3*	*FGFR3*	*IL21R*	*MKL1*	*NUP107*	*RANBP2*	*SSX2*	*USP6*
ARNT	*CCNB3*	*DDX10*	*FGFR4*	*IL3*	*MKL2*	*NUP214*	*RAP1GDS1*	*SSX4*	*VGLL3*
ASPSCR1	*CCND1*	*DDX20*	*FHIT*	*INPP5D*	*MLF1*	*NUP98*	*RARA*	*ST6GAL1*	*WASF2*
ASTN2	*CCND2*	*DEK*	*FIP1L1*	*IQCG*	*MLLT1*	*NUTM1*	*RBM15*	*STAT5B*	*WDR18*
ATF1	*CCND3*	*DMRT1*	*FLI1*	*IRF2BP2*	*MLLT10*	*NUTM2A*	*RBM6*	*STAT6*	*WDR70*

ATIC	*CD74*	*DNAJB1*	*FLNA*	*IRF4*	*MLLT11*	*NUTM2B*	*RCOR1*	*STRN*	*WHSC1*
ATP1B4	*CDH11*	*DPM1*	*FLT3*	*IRS4*	*MLLT3*	*OFD1*	*RCSD1*	*SUGP2*	*WHSC1L1*
AUTS2	*CDK5RAP2*	*DUSP22*	*FLT3LG*	*ITK*	*MLLT4*	*OLIG2*	*RET*	*SUZ12*	*WSB1*
BACH2	*CDK6*	*DUX4*	*FNBP1*	*JAK1*	*MLLT6*	*OLR1*	*RHOH*	*SYK*	*WT1*
BAG4	*CDX1*	*EBF1*	*FOSB*	*JAK2*	*MN1*	*OMD*	*RNF213*	*TACC1*	*WWTR1*
BAIAP2L1	*CDX2*	*EEFSEC*	*FOSL1*	*JAZF1*	*MNX1*	*P2RY8*	*ROS1*	*TACC2*	*XIAP*
BAZ2A	*CEBPA*	*EGFR*	*FOXO1*	*KANK1*	*MSI2*	*PAPPA*	*RPL22*	*TACC3*	*YAP1*
BCAS3	*CEBPB*	*EGR1*	*FOXO4*	*KAT6A*	*MSN*	*PATZ1*	*RPN1*	*TAF15*	*YTHDF2*
BCAS4	*CEBPD*	*EGR2*	*FOXP1*	*KAT6B*	*MUC1*	*PAX3*	*RREB1*	*TAL1*	*YWHAE*
BCL10	*CEBPE*	*EGR3*	*FRK*	*KDM5A*	*MUTYH*	*PAX5*	*RRM1*	*TAL2*	*ZBTB16*
BCL11A	*CEP170B*	*EGR4*	*FRYL*	*KIAA1524*	*MYB*	*PAX7*	*RTEL1*	*TAOK1*	*ZC3H7A*
BCL11B	*CEP85L*	*EIF4A2*	*FUS*	*KIF5B*	*MYBL1*	*PAX8*	*RUNX1*	*TBX15*	*ZC3H7B*
BCL2	*CHD6*	*ELF4*	*GAS5*	*KMT2A*	*MYC*	*PBX1*	*RUNX1T1*	*TCF12*	*ZFP64*
BCL2L1	*CHIC2*	*ELK4*	*GAS7*	*KPNB1*	*MYH11*	*PCM1*	*SARNP*	*TCF3*	*ZFPM2*
BCL3	*CHMP2B*	*ELL*	*GATA1*	*KSR1*	*MYH9*	*PDE4DIP*	*SEC31A*	*TCL1A*	*ZFYVE19*
BCL6	*CHST11*	*ELN*	*GIT2*	*LASP1*	*MYO18A*	*PDGFB*	*SEPT2*	*TCTA*	*ZMIZ1*
BCL9	*CIC*	*EML1*	*GLI1*	*LCK*	*MYO1F*	*PDGFRA*	*SEPT5*	*TEAD1*	*ZMYM2*
BCOR	*CIITA*	*EML4*	*GOSR1*	*LCP1*	*NAB2*	*PDGFRB*	*SEPT6*	*TEAD2*	*ZMYND11*
BCR	*CLP1*	*EP300*	*GOT1*	*LGR5*	*NAPA*	*PER1*	*SEPT9*	*TEAD3*	*ZNF207*
BDNF	*CLTC*	*EP400*	*GPR128*	*LHFP*	*NBEAP1*	*PHF1*	*SERPINE1*	*TEAD4*	*ZNF384*
BICC1	*CLTCL1*	*EPC1*	*GPR34*	*LHX2*	*NBR1*	*PHF23*	*SERPINF1*	*TEC*	*ZNF444*
BIRC3	*CMKLR1*	*EPOR*	*GRHPR*	*LHX4*	*NCOA1*	*PICALM*	*SET*	*TENM1*	*ZNF521*
BIRC6	*CNBP*	*EPS15*	*GRID1*	*LINC00598*	*NCOA2*	*PIM1*	*SETBP1*	*TET1*	*ZNF585B*
BRAF	*CNOT2*	*ERBB3*	*GTF2I*	*LINC00982*	*NCOA3*	*PLAG1*	*SFPQ*	*TFE3*	*ZNF687*
BRD1	*CNTRL*	*ERC1*	*H2AFX*	*LMBRD1*	*NDE1*	*PML*	*SH3D19*	*TFG*	
BRD3	*COG5*	*ERCC1*	*HAS2*	*LMO1*	*NF1*	*POM121*	*SH3GL1*	*TFPT*	
BRD4	*COL1A1*	*ERG*	*HEY1*	*LMO2*	*NFATC2*	*POU2AF1*	*SIK3*	*TFRC*	

Supplementary Table S2: Table to show the filter settings for OGM according to recommendations from Bionano.

	Filter	Setting
Filter by SV Type	Insertion	0
	Deletion	0
	Inversion	0.7
	Duplication	-1
	Intra-fusion	0.05
	Inter-translocation	0.05
General SV Filters	SV masking filter	Non-Masked Structural Variants Only
	VAF filter min	0
	VAF filter max	1
Variant Annotation Filters	SV in less than or equal to this % of the control db samples with the same enzyme	0
	SV self-molecule check	SV found in self-molecules
	Self-molecule count	5
	SV in less than or equal to this % of the control db samples	0
	SV chimerical score filter	Show Not Failing Chimeric Score
	SV overlapping genes filter	All SVs
Copy Number Variant Filters	Copy number variant type	All
	Copy number variant confidence	0.99
	Copy number variant minimum size (bp)	500,000
	Copy number variant masking filter	Non-Masked Copy Number Variants Only
Aneuploidy Filters	Aneuploidy type	All
	Aneuploidy confidence	0.95
AOH/LOH Filters	AOH/LOH minimum size (bp)	25,000,000

Supplementary Table S3: Translocations and intra-chromosomal fusions in the NB1691 neuroblastoma cell line detected by OGM

	Location	Overlap Gene(s)	Confidence	Self-Molecule Count
**Inter-Chromosomal Translocations**	t(1;6)(p22.2;q15)		0.05	11
	t(2;3)(p24.3;q26.2)		0.05	35
	t(2;3)(p24.3;q26.2)		0.06	33
	t(2;3)(p24.3;q26.2)	*AK093525*	0.26	58
	t(2;3)(p24.3;q26.2)	*AK093525*	0.52	61
	t(5;12)(q15;q15)	*CCT*	0.33	14
	t(2;12)(p24.3;q14.1)	*BC035112;MARCH9*	0.96	69
	t(2;12)(p24.3;q15)	*BC035112*	0.93	70
	t(2;12)(p24.3;q15)		0.10	54
	t(2;12)(p24.3;q15)		0.15	54
	t(2;12)(p24.3;q15)	*NBAS;RAP3IP*	0.59	70
	t(2;12)(p24.3;q15)	*NBAS;RAP3IP*	0.76	69
	t(2;12)(p24.3;q15)	*NBAS*	0.48	61
	t(2;12)(p24.3;q15)	*NBAS*	0.80	69
	t(2;12)(p24.3;q15)	*NBAS;CNOT2*	0.28	33
	t(2;12)(p24.3;q15)	*NBAS;CNOT2*	0.74	78
	t(2;12)(p24.3;q15)	*NBAS;KCNMB4*	0.71	84
	t(2;12)(p24.3;q15)	*NBAS;KCNMB4*	0.84	77
	t(2;12)(p24.3;q13.3)	*DDX1*	0.30	84
	t(2;12)(p24.3;q15)	*FRS2*	0.29	51
	t(2;12)(p24.3;q15)	*FRS2*	0.39	52
	t(2;12)(p24.3;q15)		0.85	88
	t(2;12)(p24.3;q15)	*KCNMB4*	0.71	60
	t(2;12)(p24.3;q15)	*CPM*	0.12	53
	t(2;12)(p24.3;q15)	*PTPRR*	0.75	66
	t(2;12)(p24.3;q15)	*PTPRR*	0.23	38
	t(2;12)(p24.3;q15)	*PTPRR*	0.34	41
	t(2;12)(p23.3;q13.3)	*FAM179A*	0.19	106
	t(2;12)(p23.3;q13.3)	*FAM179A*	0.38	143
	t(2;12)(p23.3;q13.3)	*FAM179A*	0.43	127
	t(2;12)(p23.3;q13.3)	*FAM179A*	0.43	183
	t(2;12)(p23.3;q13.3)	*FAM179A*	0.59	160
	t(2;12)(p23.2;q14.1)	*ALK;AVIL*	0.32	200
	t(2;12)(p23.2;q14.1)	*ALK;AVIL*	0.32	202
	t(2;12)(p23.2;q14.1)	*ALK;AVIL*	0.36	191
	t(2;12)(p23.2;q14.1)	*ALK;AVIL*	0.51	234
	t(2;12)(p23.2;q14.1)	*ALK;AVIL*	0.51	238
	t(2;12)(p23.2;q14.1)	*ALK;AVIL*	0.68	190
	t(2;12)(p23.2;q14.1)	*ALK;AVIL*	0.71	174
	t(2;12)(p23.2;q14.1)	*ALK;AVIL*	0.36	173
	t(2;12)(p23.2;q14.1)	*ALK;AVIL*	0.44	173
	t(2;12)(p23.2;q14.1)	*ALK;AVIL*	0.55	227
	t(2;12)(p23.2;q21.1)	*ALK;TSPAN8*	0.06	71

	t(2;12)(p23.2;q21.1)	*ALK;TSPAN8*	0.08	75
	t(2;12)(p16.3;q15)		0.11	153
	t(2;12)(p16.3;q15)		0.06	120
	t(2;12)(p16.3;q15)		0.11	139
**Intra-Chromosomal Fusions**	inv(5)(p15.q15)	*CTNND2*	0.05	11
	inv(5)(p15.1p13.3)		0.09	12
	inv(5)(p14.3q12.3)		0.08	20
	inv(5)(p14.3q12.3)		0.09	13
	inv(5)(p15.31q13.31)		0.09	7
	inv(5)(p15.31q33.3)	*ADCY2;CCNJL*	0.07	11
	inv(5)(p15.31q33.3)	*ADCY2;CCNJL*	0.08	11
	inv(2)(p24.3)	*NBAS*	0.78	254
	inv(2)(p24.3)		0.60	74
	inv(2)(p24.3p23.2)	*ALK*	0.51	70
	inv(2)(p24.3p23.2)	*ALK*	0.65	71
	inv(2)(p24.3p23.2)	*MYCN*	0.06	61
	inv(2)(p24.3p23.2)	*MYCN*	0.08	61
	inv(2)(p23.2p16.3)	*NRXN1*	0.33	138
	inv(2)(p23.2p16.3)	*NRXN1*	0.39	147
	inv(2)(p23.1p16.3)	*NRXN1*	0.05	97
	inv(2)(p23.1p16.3)	*NRXN1*	0.22	114
	inv(12)(q13.13q14.1)	*KRT86*	0.05	49
	inv(12)(q13.13q14.1)	*KRT86*	0.14	65
	inv(12)(q13.13q14.1)	*KRT86*	0.15	181
	inv(12)(q13.13q14.1)	*KRT86*	0.23	220
	inv(12)(q13.13q14.1)	*KRT86*	0.13	72
	inv(12)(q13.3q15)	*ARHGEF25*	0.05	64
	inv(12)(q14.1q15)	*AVIL*	0.20	56
	inv(12)(q14.1q24.22)		0.05	199
	inv(12)(q14.1q24.22)		0.05	202
	inv(12)(q14.1q24.22)		0.05	249
	inv(12)(q14.1q24.22)		0.22	112
	inv(12)(q14.1q24.22)	*RNFT2*	0.05	170
	inv(12)(q14.1q24.22)	*RNFT2*	0.07	145
	inv(12)(q14.1q24.22)	*KCNMB4*	0.13	51
	inv(12)(q14.1q15)	*TSPAN8*	0.09	74
	inv(12)(q14.1q15)	*CPM*	0.15	70
	inv(12)(q21.1q15)	*SLC35E3*	0.05	447
	inv(12)(q21.1q15)	*SLC35E3*	0.06	726
	inv(12)(q21.1q15)	*SLC35E3*	0.22	815
	inv(12)(q21.1q15)	*SLC35E3*	0.05	537
	inv(12)(q21.1q15)	*SLC35E3*	0.34	812
	inv(12)(q21.1q15)	*SLC35E3*	0.67	835

Supplementary Table S4: RNA fusion panel results. A confidence score should be > 0.4 with > 3 supporting reads

Cell Line	Fusion	Score	Reads	Partner 1	Partner 2
**NB1691**	*PTPRR*::*BEST3*	0.523	9	Ch12 *PTPRR* 71147970	Ch12 *BEST3* 70049592
**SH-SY5Y**	*EXOC4*::*PVT1*	0.441	6	Ch7 *EXOC4* 133059753	Ch8 *PVT1* 129108761

Supplementary Table S5: Translocations and Intra-chromosomal fusions in the NBLW neuroblastoma cell line detected by OGM

	Location	Overlap Gene(s)	Confidence	Self-Molecule Count
**Inter-Chromosomal Translocations**	t(16;17)(p13.3;q21.31)	*CRAMP1L;HDAC5*	0.40	15
	t(2;6)(p23.3;p21.2)		0.19	15
	t(2;6)(p23.3;p21.2)		0.68	13
	t(2;6)(p25.3;p21.2)		0.42	17
	t(2;6)(p25.3;p21.2)		0.46	19
	t(2;19)(p24.3;q13.43)	*NBAS;BC036412*	0.09	23
	t(2;19)(p24.3;q13.43)	*NBAS;BC036412*	0.35	26
	t(2;19)(p24.3;q13.43)	*NBAS*	0.91	86
	t(2;19)(p24.3;q13.43)	*NBAS;ZCAN4*	0.63	38
	t(2;19)(p24.3;q13.43)		0.11	36
	t(2;19)(p24.3;q13.43)		0.1	47
	t(2;19)(p24.3;q13.43)		0.94	43
	t(2;19)(p24.3;q13.43)		0.36	26
	t(2;19)(p24.3;q13.43)	*ZNF274*	0.43	22
	t(2;19)(p24.3;q13.43)	*ZNF274*	0.52	23
	t(1;2)(q44;p25.3)		0.32	20
	t(1;2)(q44;p25.3)		0.65	21
	t(1;2)(p21.3;p25.2)		0.76	17
**Intra-Chromosomal Fusions**	inv(2)(p24.3)	*NBAS*	0.34	40
	inv(2)(p24.3)		0.20	634
	inv(2)(p24.3)		0.34	987
	inv(2)(p24.3)		0.15	400
	inv(2)(p24.3)		0.16	726
	inv(2)(p24.3)		0.76	1137
	inv(2)(p24.3)		0.91	1057
	inv(2)(p24.3)		0.95	945
	inv(2)(p24.3)	*NBAS*	0.25	405
	inv(2)(p24.3)	*NBAS*	0.90	406
	inv(2)(p24.3)	*NBAS*	0.12	56
	inv(2)(p25.3p24.3)		0.90	16

	inv(2)(p25.2)	*LOC150622*	0.50	17
	inv(2)(p25.2p24.1)		0.27	16
	inv(2)(p25.2p24.1)		0.29	10
	inv(2)(p25.2p24.1)	*LOC150622*	0.16	20
	inv(2)(p25.2p24.2)		0.13	16
	inv(2)(p25.2p24.2)		0.58	16

Supplementary Figure S1: NBLW cell line A) OGM Circos plot view. B) OGM Circos plot view of chromosomes 2 and 19 showing potential chromoplexy. C) OGM Whole Genome View. OGM SV/SNV Key: Green, insertion; Orange, deletion; Light blue, inversion; Purple, duplication; Pink, Intra-chromosomal fusion or Inter-chromosomal Translocation; Yellow, LOH region; Dark blue, CNV Gain; Red, CNV Loss. D) SNP array analysed using Nexus software. i) log2 ratio ii) B allele frequency. *MYCN* amplicon highlighted in B –D.

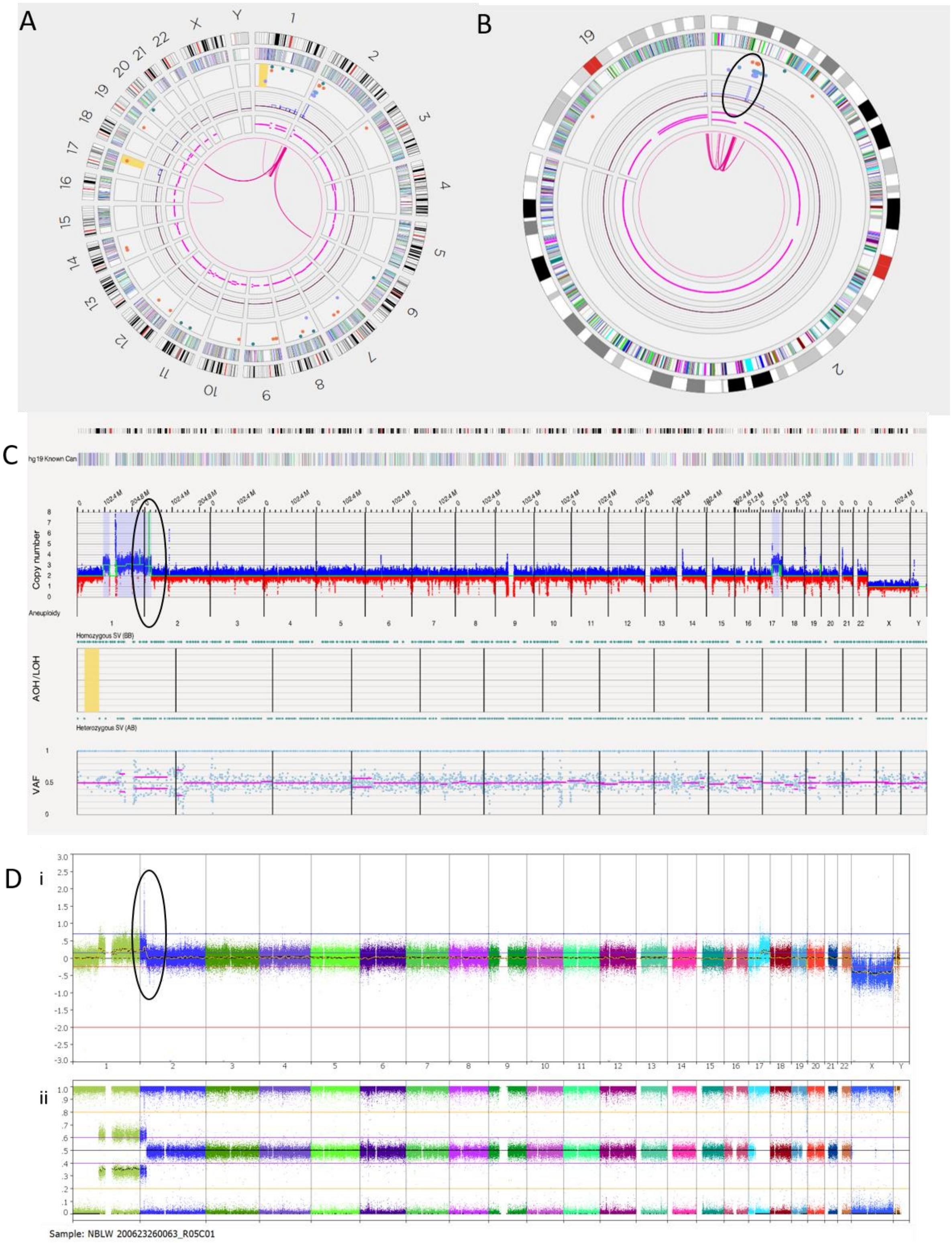

Supplementary Figure S2: Neuroblastoma Tumour 2. A) OGM Circos plot view of. 2 B) WGS Circos plot view. C) OGM Whole Genome View. OGM SV/SNV Key: Green, insertion; Orange, deletion; Light blue, inversion; Purple, duplication; Pink, Intra-chromosomal fusion or Inter-chromosomal Translocation; Yellow, LOH region; Dark blue, CNV Gain; Red, CNV Loss. D) SNP array analysed using Nexus software. i) log2 ratio ii) B allele frequency.

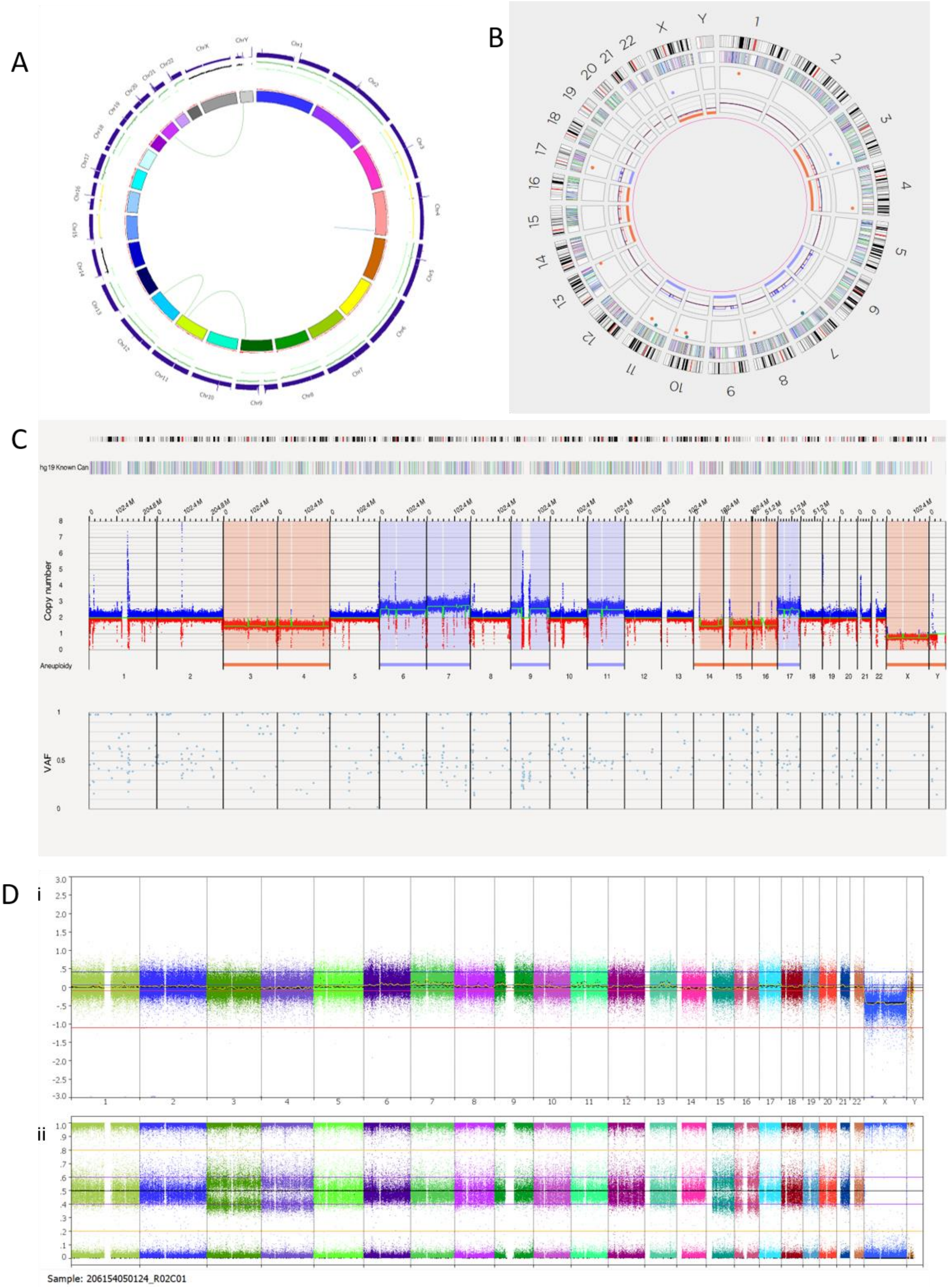

Supplement: Supplementary file 1 [file cancers-15-05233-s001.zip › cancers-2657680-supplementary.pdf]
